# Early probiotic supplementation with *B. infantis* in breastfed infants leads to persistent colonization at 1 year

**DOI:** 10.1038/s41390-020-01350-0

**Published:** 2021-03-24

**Authors:** Claire E. O’Brien, Anna K. Meier, Karina Cernioglo, Ryan D. Mitchell, Giorgio Casaburi, Steven A. Frese, Bethany M. Henrick, Mark A. Underwood, Jennifer T. Smilowitz

**Affiliations:** 1 Department of Food Science and Technology, University of California Davis, Davis, CA USA; 2 Foods for Health Institute, University of California Davis, Davis, CA USA; 3 Evolve BioSystems, Inc., Davis, CA USA; 4 Prescient Metabiomics, 600 Faraday Ave, Carlsbad, CA 92008 USA; 5 Department of Nutrition, University of Nevada, Reno, Reno, NV 89557 USA; 6 Department of Food Science and Technology, University of Nebraska, Lincoln, NE USA; 7 Department of Pediatrics, University of California Davis Children’s Hospital, Sacramento, CA USA

## Abstract

**Background:**

Recent studies have reported a dysfunctional gut microbiome in breastfed infants. Probiotics have been used in an attempt to restore the gut microbiome; however, colonization has been transient, inconsistent among individuals, or has not positively impacted the host’s gut.

**Methods:**

This is a 2-year follow-up study to a randomized controlled trial wherein 7-day-old infants received 1.8 × 10^10^ colony-forming unit *Bifidobacterium longum* subsp. *infantis* (*B. infantis*) EVC001 (EVC) daily for 21 days or breast milk alone (unsupplemented (UNS)). In the follow-up study, mothers (*n* = 48) collected infant stool at 4, 6, 8, 10, and 12 months postnatal and completed the health-diet questionnaires.

**Results:**

Fecal *B. infantis* was 2.5–3.5 log units higher at 6–12 months in the EVC group compared with the UNS group (*P* < 0.01) and this relationship strengthened with the exclusion of infants who consumed infant formula and antibiotics. Infants in the EVC group had significantly higher *Bifidobacteriaceae* and lower *Bacteroidaceae* and *Lachnospiraceae* (*P* < 0.05). There were no differences in any health conditions between the two groups.

**Conclusions:**

Probiotic supplementation with *B. infantis* within the first month postnatal, in combination with breast milk, resulted in stable colonization that persisted until at least 1 year postnatal.

**Impact:**

- A dysfunctional gut microbiome in breastfed infants is common in resource-rich nations and associated with an increased risk of immune diseases.
- Probiotics only transiently exist in the gut without persistent colonization or altering the gut microbiome.
- This is the first study to show that early probiotic supplementation with *B. infantis* with breast milk results in stable colonization of *B. infantis* and improvements to the gut microbiome 1 year postnatal.
- This study addresses a key gap in the literature whereby probiotics can restore the gut microbiome if biologically selected microorganisms are matched with their specific food in an open ecological niche.

## Introduction

Human milk delivers a wide spectrum of biologically active molecules that aid in the development and maturation of the gut and the innate and adaptive immune systems and support the growth of protective intestinal microbiota. Specifically, human milk oligosaccharides (HMOs), the third most abundant component in human milk (~10–20 g/L),^1,2^ are a group of complex sugars that are nondigestible by the human infant and support the competitive growth of protective bifidobacterial strains within the intestine.^3,4^ In particular, the natural colonization of a protective subspecies of *Bifidobacterium*, *Bifidobacterium longum* subsp. *infantis* (*B. infantis*), unlike other bifidobacterial species in breastfed infants, is based on its genetic capabilities to bind, transport, and ferment HMOs into lactate and acetate.^4,5^ These fermentative products maintain a lower pH of the intestinal milieu, support the transport of these compounds into the intestinal epithelium for use by the host,^6^ create an undesirable environment for potential pathogens,^7^ and prevent the infiltration of toxic molecules produced by pathogenic bacteria by upregulating intestinal barrier function and inhibiting proinflammatory and apoptotic responses.^8^

Historically, the gut of the breastfed infant was dominated by a near monoculture of *Bifidobacterium* until the cessation of breastfeeding.^9^ However, findings from Henrick et al.^10^ reported a generational loss of *Bifidobacterium* in breastfed infants from resource-rich nations within the past 100 years accompanied by higher levels of enteropathogens and higher fecal pH.^10^ The reduction in *Bifidobacterium* and increase in potential pathogens in the infant gut microbiome are likely a result of the unintended consequences of antibiotic use, infant formula feeding,^11^ and cesarean section deliveries,^12^ all of which have been implicated in the increased risk for allergic and autoimmune diseases prevalent in resource-rich nations.^13–15^ Colonization of a dysfunctional gut microbiome in early infancy during the critical window of immune system development is reported to increase the risk for the development of immune disease later in life.^16^

We previously published findings from the IMPRINT Study in which healthy, term, breastfed infants supplemented with 1.8 × 10^10^ colony-forming units (CFUs) of *B*. *infantis* EVC001 per day for 21 consecutive days starting on day 7 postnatal demonstrated persistent colonization of fecal *B. infantis* 1 month post supplementation. Given the diversity among *B. infantis* strains,^5,17^ we selected *B. infantis* EVC001 because we knew this strain had the full cassette of genes needed to completely digest all HMOs from human milk. Supplementation with *B*. *infantis* EVC001 was well tolerated^18^ and increased fecal *Bifidobacteriaceae* by 79% and reduced enteropathogens by 80%, decreased fecal HMOs by 10-fold (consistent with increased HMO consumption by gut microbes), and increased fecal lactate and acetate by 2-fold, resulting in a decrease in fecal pH by 1 log unit.^19^ Intestinal colonization of *B. infantis* persisted 1 month post supplementation. These results are unprecedented as probiotics have only been found to transiently exist in the gut during supplementation in infants, without showing persistent colonization in most individuals or altering the gut microbiome composition in adults.^20^ In the follow-up study reported herein, infants who completed the IMPRINT Study at 2 months of age were followed up at 4, 6, 8, 10, 12, 18, and 24 months postnatal. The aims of this follow-up study were to determine if *B. infantis* colonization persisted up to 1 year postnatal and identify differences in reported health outcomes between *B. infantis* EVC001 supplemented and unsupplemented (UNS) infants.

## Methods

### Subjects and design

The details of the main 2-month-long IMPRINT Study are reported elsewhere.^18^ Briefly, mother–infant dyads were recruited in the Davis and Sacramento metropolitan region of Northern California. Mothers received either lactation support or lactation support and 1.8 × 10^10^ CFU of *B. infantis* EVC001 (ATCC SD-7035; manufactured by Evolve BioSystems, Inc.) to feed their infants daily from days 7 to 27 postnatal. *Bifidobacterium infantis* EVC001 was delivered as 156 mg of live bacteria (1.8 × 10^10^ CFU) diluted in 469 mg of lactose as an excipient. Mothers were trained by lactation consultants to mix the *B. infantis* EVC001 powder with 5 mL of expressed breast milk and feed the mixture to their infant using a feeding syringe. The probiotic was stored at −20 °C by the mothers during the study. Upon completing the parent trial when their infants were ~2 months of age, participants were offered the opportunity to enroll in two independent follow-up studies: follow-up #1, which was designed to determine if *B. infantis* persisted up to 1 year postnatal; and follow-up #2, which was designed to determine if *B. infantis* supplementation early in life was protective against the development of health conditions at 18 and 24 months postnatal. In the follow-up #1 study, mothers completed a paper questionnaire about their infants’ health and diet at 4, 6, 8, 10, and 12 months postnatal and collected one matching infant fecal sample at each time point. In the follow-up #2 study, mothers completed an online questionnaire about their infants’ health and diet at 18 and 24 months postnatal. The study and methods were approved by the UC Davis Institutional Review Board, and the study was registered at Clinicaltrials.gov (NCT02457338). All mothers provided written informed consent to participate in every aspect of the study.

### Questionnaires

#### Follow-up studies

Mothers who completed the parent IMPRINT Study were invited to enroll in two different follow-up studies: follow-up #1 and follow-up #2. In follow-up #1, upon providing written informed consent, mothers completed up to five paper questionnaires that coincided with the collection of their infants’ stool at 4, 6, 8, 10, and 12 months postnatal (Supplementary File 1 (online)). In follow-up #2, upon providing email informed consent, mothers completed up to two questionnaires (without stool collection) at 18 and 24 months postnatal (Supplementary File 2 (online)). The questionnaires that were used in both follow-up studies prompted mothers to answer questions about their infants’ health and diet. Specifically, in follow-up #1, mothers were prompted to report on their infants’ health and diet over the past 2 months (either since completing the parent IMPRINT Study or since completing the previous questionnaire). In follow-up #2, mothers were prompted to report their infants’ health and diet over the past 6 months at 18 and 24 months postnatal. In both the follow-up #1 and #2 studies, mothers were asked questions about their infants’ dietary patterns (intake of breast milk, infant formula, and solid foods), use of medications, supplements and vitamins, illnesses, sick doctor visits, hospitalizations, antibiotic (oral/IV) usage, and probiotic intake.

In follow-up #1 only, mothers were asked if their infants were ever diagnosed with common infant health illnesses and conditions by a healthcare professional and infant age at diagnosis. The answer options were “diagnosed” or “not diagnosed.” Mothers were asked to report the frequency of common infant conditions and illnesses. The answer options were “never,” “sometimes,” “often,” “very often,” “unsure,” and “refuse.” When mothers answered “sometimes,” “often,” or “very often,” they were prompted to rate the severity of the gastrointestinal symptoms from 1 to 10, with 1 as the least severe and 10 as the most severe.

In follow-up #2, mothers were asked if their infants had experienced and were diagnosed with any allergies, wheezing, asthma, eczema, gastroesophageal reflux disease, and lactose intolerance. The answer options were “yes,” “no,” “unsure,” and “refuse.” When mothers answered “yes,” they were prompted to report the number of times their infants had experienced and if they had been diagnosed with common infant illnesses and conditions.

### Samples

#### Follow-up #1

Fecal samples were collected at home at 4, 6, 8, 10, and 12 months postnatal. Fecal samples were stored in participants’ home freezers and transferred on dry ice to a −80 °C freezer for storage prior to DNA extraction. All individuals who processed and analyzed the samples were blinded to treatment allocation.

### Molecular methods and analyses

As previously described,^19^ total DNA was extracted from ~100 mg of feces, using the Zymo Fecal DNA Miniprep Kit according to the manufacturer’s instructions (Zymo Research, Irvine, CA). Negative controls to detect kit contamination were included and failed to produce visible PCR bands in an agarose gel but were analyzed as quality controls. Samples were subjected to 16S ribosomal RNA (rRNA) gene sequencing as previously described.^19^ Quantification of the total *B. infantis* was performed by quantitative real-time PCR using Blon_2348 sialidase gene primers Inf2348F (5′-ATA CAG CAG AAC CTT GGC CT-3′), Inf2348_R (5′-GCG ATC ACA TGG ACG AGA AC-3′), and Inf2348_P (5′-/56-FAM/TTT CAC GGA /ZEN/TCA CCG GAC CAT ACG/3lABkFQ/-3′). The *Blon_2348* gene is found in all *B. infantis* strains including EVC001. The primer and probe sequence specificity has been previously described.^21^ Each reaction contained 10 μL of 2× TaqMan Universal Master Mix II with UNG master mix (Thermo Fisher Scientific, Waltham, MA), 0.9 µM of each primer, 0.25 µM probe, and 5 μL of template DNA. Thermal cycling was performed on a QuantStudio 3 Real-Time PCR System (Thermo Fisher Scientific, Waltham, MA) and consisted of an initial UNG activation step of 2 min at 50 °C, followed by a 10-min denaturation at 95 °C, succeeded by 40 cycles of 15 s at 95 °C and 1 min at 60 °C. All samples were run in duplicate with a standard curve on each plate. Quantification of *B. infantis* was determined (CFU/g stool) using a standard curve of genomic DNA derived from a pure culture of *B. infantis* EVC001 using CFU counts and normalized for input stool wet weight.^22^ Standard curve genomic DNA was extracted from 1 mL aliquots of *B. infantis* EVC001 grown anaerobically at 37 °C for 16 h in deMann Rogosa Sharpe (MRS) medium (BD Biosciences, San Jose, CA) supplemented with 0.05% l-cysteine HCl. CFU counts of the 16-h *B. infantis* EVC001 culture were determined by serial dilution in 0.9% NaCl on MRS agar plates containing 0.05% l-cysteine HCl. Plates were incubated anaerobically at 37 °C for 48 h, then counted, and the CFU/mL value was calculated.

### 16S rRNA bioinformatics analysis

Sequences were analyzed using QIIME 1.9.1 (10.1038/nmeth.f.303). Open-reference operational taxonomical unit (OTU) picking was performed using UCLUST at 97% identity against the Greengenes database (v.13_8) (10.1128/AEM.03006-05), and chimera filtering was checked as part of the QIIME pipeline using USEARCH 6.1.^23^

A representative set of sequences was taken for each OTU and taxonomic classification was performed using UCLUST consensus taxonomy in QIIME. Representative sequences were then aligned using PyNAST (https://biocore.github.io/pynast/) to the Greengenes core reference alignment and a phylogenetic tree was built using FastTree.^24^ After quality filtering, a mean of 26,354 (±8830 [SD]) and a median of 27,646 reads were obtained per sample. Several multivariate linear modeling analyses (https://huttenhower.sph.harvard.edu/maaslin/) were computed to compare groups of samples at the family and genus levels, using the subject as a random effect to account for time and other clinical metadata, including treatment status, delivery mode, and feeding as fixed effects. Multivariate Association with Linear Models 2 (MaAsLin2) was run with a false-discovery rate (FDR) of 0.05, a minimum of 0.0001 for feature relative abundance filtering, and a minimum of 0.01 for feature prevalence filtering. Fixed effects used in the MaAsLin2 model include any use of the following by the infant: antibiotics, probiotics, probiotics containing *B. infantis*, infant formula, solid food, and breast milk. In addition, the model included delivery mode, supplementation allocation. Subject ID was used as a random effect and time was used as a continuous variable. *P* values were adjusted via FDR (*Q* values) and considered significant if *Q* value < 0.25. Raw data are accessible under the accession number PRJNA670448.

### Diversity analysis

Rarefaction curves were computed to estimate the distribution of the identified OTUs at a depth of 1538 sequences/sample. Alpha-diversity was computed using the Shannon diversity index in QIIME. A nonparametric two-sample *t* test was used to compare alpha-diversity according to treatment status using Monte Carlo permutations (*n* = 999). Beta-diversity was computed using UniFrac distances and a dissimilarity matrix was constructed to estimate the global OTUs differences among samples and visualized via a principal coordinate analysis. A permutational multivariate analysis of variance using distance matrices (adonis) was used to assess OTU differences between treatments and the effect size (*R*^2^) of colonization by EVC001. *P* values for the principal coordinate analysis panel were computed using *F* tests based on sequential sums of squares from permutations of the raw data.

### Statistics

The Mann–Whitney *U* test was used to compare mean ranks for fecal *B. infantis* between EVC and UNS groups at each time point during the first follow-up period (4, 6, 8, 10, and 12 months postnatal). The Mann–Whitney *U* test was performed on (1) all infants, (2) infants who had not used any infant formula, antibiotics, or probiotics since completing the parent IMPRINT Study (2 months postnatal), and (3) infants who used infant formula or antibiotics at 6 months postnatal (or time-point closest to 6 months). Because solid foods are commonly introduced to infants by 6 months of age, solid food consumption was not excluded in any of the analyses. Infant weight was measured at each study visit using a Pediatric Tanita digital scale and mean ranks for infant weight were compared between EVC and UNS groups using Mann–Whitney *U* test. The significance level for all Mann–Whitney *U* test analyses was set at an *α* 0.05 with a Bonferroni adjustment using the two-tailed exact test statistic, which is appropriate for small, unbalanced, or poorly distributed data. Mean ranks for frequency ordinal data and for severity continuous data were compared between EVC and UNS groups using Mann–Whitney *U* test. Categorical data that resulted in the answers “yes,” “no,” “unsure,” or “refuse” were analyzed using a two-sided Fisher’s exact test with an *α* 0.05 with a Bonferroni adjustment, whereby “unsure” and “refuse” responses were excluded from the analysis. SPSS version 25 was used for these analyses.

## Results

### Follow-up #1

**Fig. 1:**
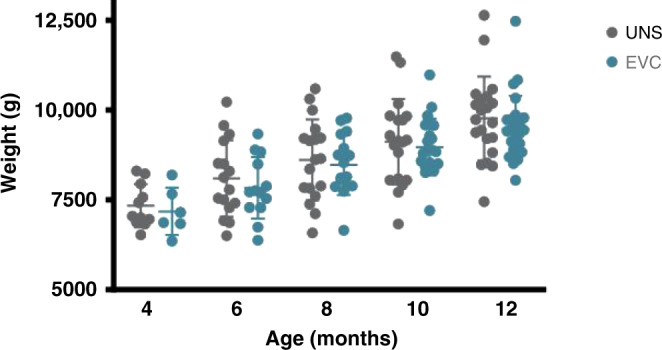
Infant weight across time and between treatment groups for all infants. Sample size is not consistent with Table 3 due to missed weights for EVC: day 120, *n* = 6; day 300, *n* = 22; day 365, *n* = 25.

Of the 68 mothers enrolled in the parent IMPRINT Study, 48 mothers enrolled in the follow-up #1 study. Of these 48 mother–infant dyads, *n* = 22 had received the UNS treatment and *n* = 26 had received the EVC treatment. There was a significantly higher number of primiparous women in the EVC group than in the UNS group (*P* < 0.01) (Table 1). There were no other differences in demographic, labor, delivery, and health history characteristics between the two groups. Infants enrolled in the EVC group were born at a younger gestational age than infants enrolled in the UNS group (*P* < 0.05) (Table 2); however, all infants were full term at birth. A detailed description of infants’ diet, intake of antibiotics and probiotics, and exposure to other infants via daycare are reported in Table 3. There were no differences in the number of infants who consumed breast milk; breast milk and infant formula; infant formula without breast milk; solid foods; used antibiotics or probiotics; or were enrolled in daycare at any time point (Table 3). There was no difference in weight between groups across time (Fig. 1).

**Table 1:** Maternal demographics, labor, delivery, and health history.

Characteristics	UNS (*n* = 22)		EVC (*n* = 26)	
	Mean	SD	Mean	SD
Maternal age at enrollment (years)	31.0	3.4	33	4.7
Prepregnancy BMI	24.5	3.1	26.2	3.5
Pregnancy weight gain (kg)	31.1	7.7	33.7	11.8
Hours in labor	22.0	26.0	11.3	12.6
Ruptured membranes prior to birth (h)	12.4	19.2	7.1	12.0
Number of pregnancies	2.0	1.5	2.8	1.7*
Number of live births	1.5	1.0	2.2	1.1**
Parity, % (*n*)				
Primiparous	77.3% (17)		34.6% (9)**	
Multiparous	22.7% (5)		65.4% (17)	
Mode of delivery, % (*n*)				
Vaginal	63.6% (14)		69.2% (18)	
Vaginal water birth	18.2% (4)		0% (0)	
C-section, emergent	13.6% (3)		15.4% (4)	
C-section, elective	4.5% (1)		15.4% (4)	
Ethnicity, % (*n*)				
Not hispanic	90.9% (20)		76.9% (20)	
Hispanic	9.1% (2)		23.1% (6)	
Race, % (*n*)				
Asian	4.5% (1)		0% (0)	
Black	4.5% (1)		0% (0)	
White	81.8% (18)		73.1% (19)	
Other	0% (0)		7.7% (2)	
2 or More races	9.1% (2)		19.2% (5)	
Education, % (*n*)				
Some college, no degree, or AA degree	13.6% (3)		19.2% (5)	
Bachelor’s degree (BA or BS)	36.4% (8)		34.6% (9)	
Master’s, professional, or doctorate degree	50% (11)		46.2% (12)	
Antibiotic use during labor, % (*n*)				
Yes	18.2% (4)		26.9% (7)	
No	81.8% (18)		73.1% (19)	
Gestational diabetes mellitus positive diagnosis, % (*n*)				
Yes	9.1% (2)		7.7% (2)	
No	90.9% (20)		92.3% (24)	
Group B *Streptococcus* (GBS) colonization positive diagnosis, % (*n*)				
Yes	22.7% (5)		30.8% (8)	
No	77.3% (17)		69.2% (18)	
Any allergy diagnosis in past 10 years, % (*n*)				
Yes	36.4% (8)		26.9% (7)	
No	63.6% (14)		73.1% (19)	
Asthma diagnosis in past 10 years, % (*n*)				
Yes	22.7% (5)		7.7% (2)	
No	77.3% (17)		92.3% (24)	
Hay fever diagnosis in past 10 years, % (*n*)				
Yes	0% (0)		7.7% (2)	
No	100% (22)		92.3% (24)	
Autoimmune disease diagnosis in past 10 years, % (*n*)				
Yes	0% (0)		15.4% (4)	
No	100% (22)		84.6% (22)	
Impaired glucose tolerance in past 10 years, % (*n*)				
Yes	0% (0)		0% (0)	
No	100% (22)		100% (26)	

*P < 0.05.

**P < 0.01 for differences between treatment groups.

**Table 2:** Infant characteristics.

Infant Characteristics	UNS (*n* = 22)		EVC (*n* = 26)	
	Mean	SD	Mean	SD
Gestational age at birth (weeks)	40.2	1.0	39.5	1.3*
Birth weight (g)	3669.2	587.8	3448.8	396.3
Birth length (cm)	51.1	2.4	50.5	2.2
Gender, % (*n*)				
Male	40.9% (9)		65.4% (17)	
Female	59.1% (13)		34.6% (9)	

*P < 0.05 for differences between treatment groups.

**Table 3:** Infant diet and environment^a^.

Month										
Feeding and environment	4		6		8		10		12	
	UNS	EVC	UNS	EVC	UNS	EVC	UNS	EVC	UNS	EVC
Total (*n*)	11	7	16	13	18	16	19	23	21	26
Breast milk^a^ (*n*)	8	6	12	9	10	10	9	13	10	12
Breast milk and infant formula^a^ (*n*)	2	1	1	4	3	4	2	5	4	5
Infant formula^a^ (*n*)	0	0	1	0	1	1	2	1	3	3
Solids (*n*)	1	0	14	9	18	16	19	23	21	26
Antibiotics (*n*)	0	0	1	0	2	1	4	4	3	4
Probiotics^b^ (*n*)	1	0	1	0	3	1	4	2	1	3
Daycare (*n*)	27%	0%	50%	23%	50%	25%	47%	35%	57%	50%

^a^Excludes infants who took antibiotics and/or probiotics.

^b^*n* = 4 infants in the EVC and *n* = 5 in the UNS consumed five different probiotic supplement products at various times during the follow-up study. Participants in the study were able to recall the product names for four of the five probiotic supplements they fed to their infants. The four probiotic products recalled contained the following microorganisms: (1) *Lactobacillus acidophilus* and *Lactobacillus helveticus* (unspecified strains), (2) *Bifidobacterium longum* and *Bifidobacterium infantis* (unspecified strains), (3) proprietary probiotic blend containing five *Lactobacillus* and five *Bifidobacterium* species, and (4) *B. infantis* EVC001 (one participant enrolled in the study found one sachet of the study probiotic in her freezer and fed it to her infant at 11 months postnatal).

**Fig. 2:**
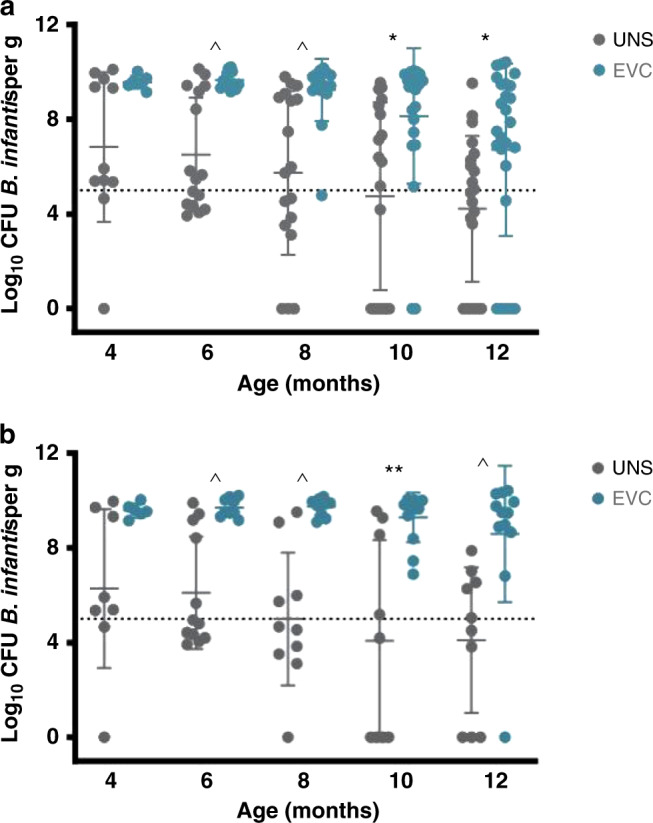
Infant fecal *B. infantis* across time and between treatment groups. **a** Inclusion of all infants. **b** In a subgroup of infants who did not receive infant formula, antibiotics, or probiotics. *P < 0.01, **P < 0.001, and ^*P* < 0.0005 for differences between treatment groups.

**Fig. 3:**
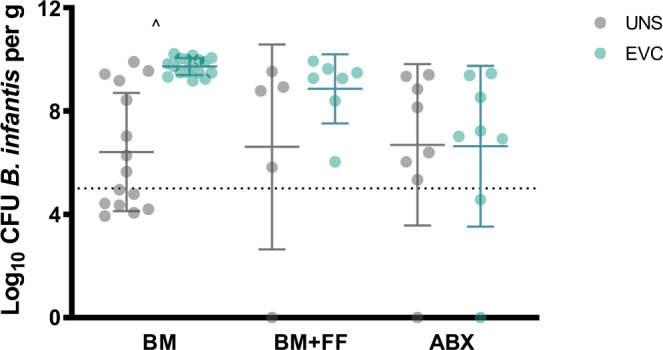
Infant fecal *B. infantis* at 6 months postnatal among three subgroups of infants based on diet and exposure to antibiotics. BM breast milk (including solids); BM + FF breast milk and formula-fed (including solids), ABX antibiotic use. ^*P* < 0.0005 for differences between treatment groups.

**Fig. 4:**
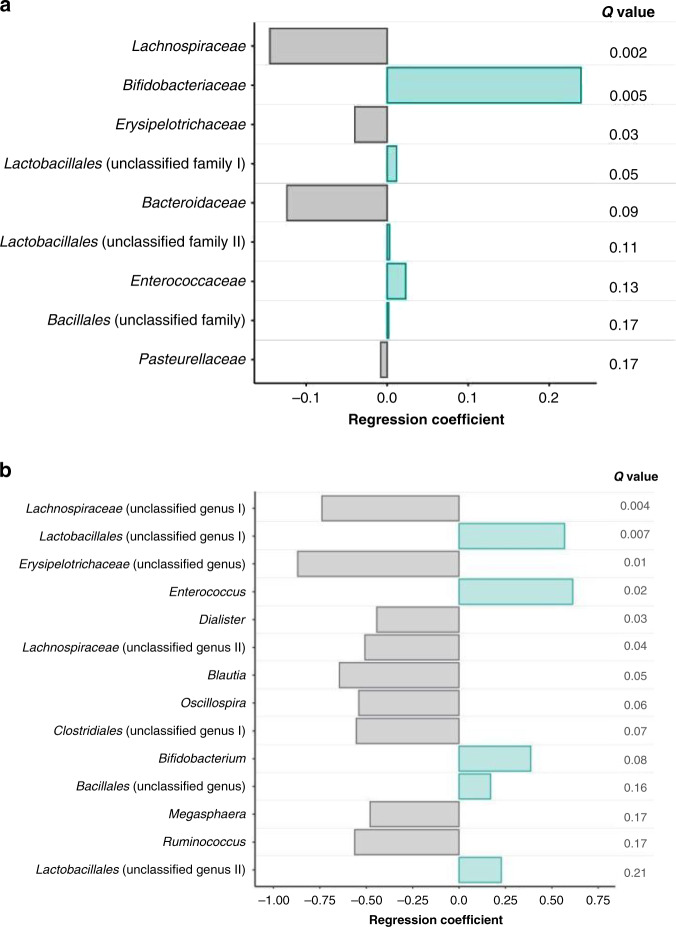
Relationships between infant fecal microbial families and treatment groups based on MaAsLin2 for all infants. **a** Family level. **b** Genus level. *P* values were adjusted via FDR (*Q* values) and considered significant if *Q* value < 0.25.

With the inclusion of all infants, fecal *B. infantis* was 2.5–3.5 logs higher at 6–12 months in the EVC group compared with the UNS group (*P* < 0.01) (Fig. 2a). In a subgroup of infants who did not receive infant formula, antibiotics, or probiotics, fecal *B. infantis* was 3.6–5.2 logs higher at 6–12 months in the EVC group compared with the UNS group (*P* < 0.001) (Fig. 2b). To further focus on these confounding variables, we conducted Mann–Whitney *U* testing on three subgroups: infants breast milk fed without intake of infant formula, antibiotics, or probiotics (BM); infants mixed fed with breast milk and infant formula without intake of antibiotics or probiotics (BM + FF); and infants exposed to antibiotics and/or additional probiotics (all feeding types) (ABX). We selected one time point, as close to 6 months as possible, when breast milk volume intake would be the highest and the introduction of solid foods would be minimal. For this analysis, for the BM subgroup, fecal *B. infantis* was 3.3 logs higher in infants in the EVC group compared to the UNS group (*P* < 0.0005). However, for both the BM + FF and ABX subgroups, fecal *B. infantis* was not different between EVC and UNS groups (Fig. 3). To further investigate how *B. infantis* supplementation influences the gut microbial composition across all time points, we used MaAsLin2, to determine if treatment altered gut microbial taxa. Infants in the EVC group had significantly higher *Bifidobacteriaceae* (*R* = 0.24, FDR-adjusted *Q* value < 0.01), *Lactobacillales* unclassified family I (*R* = 0.01, FDR-adjusted *Q* value = 0.05), *Lactobacillales* unclassified family II (*R* = 0.003, FDR-adjusted *Q* value = 0.12), *Enterococcaceae* (*R* = 0.02, FDR-adjusted *Q* value = 0.14), and *Bacillales* unclassified family (*R* = 0.002, FDR-adjusted *Q* value = 0.17) and significantly lower *Lachnospiraceae* (*R* = 0.14, FDR-adjusted *Q* value < 0.01), *Erysipelotrichaceae* (*R* = 0.04, FDR-adjusted *Q* value < 0.05), Bacteroidaceae (*R* = 0.12, FDR-adjusted *Q* value = 0.09), and *Pasteurellaceae* (*R* = 0.008, FDR-adjusted *Q* value = 0.17) compared with the UNS group (Fig. 4a) even after adjustments for infant formula, antibiotics, probiotics, delivery mode, postnatal age, and subject as a random variable. Of the taxa that were significantly different between treatments according to MaAsLin2, Mann–Whitney *U* test was used to compare differences for taxa between treatments for each time point and confirmed statistical differences for only fecal *Bifidobacteriaceae* and *Lachnospiraceae* at 6, 8, 10, and 12 months postnatal (*P* < 0.05) and *Bacteroidaceae* at 12 months postnatal (*P* < 0.01) (Table 4). The same MaAsLin2 modeling used on a family level showed higher correlation coefficients between supplementation and gut microbial composition on a genus level (Fig. 4b). The genera that were significantly different between treatments according to MaAsLin2 modeling were compared statistically at each time point using Mann–Whitney *U* test. Infants in the EVC group had significantly higher fecal *Bifidobacterium* at 6, 8, 10, and 12 months postnatal, and *Enterococcus* at 6 months postnatal and lower *Lachnospiraceae* (unclassified genus) at 6, 8, and 10 months postnatal; *Ruminococcus* at 8 months postnatal; and *Erysipelotrichaceae* (unclassified genus) at 6 and 8 months postnatal (Supplementary Table S1).

**Table 4:** Infant fecal microbial families measured by 16s rRNA amplicon sequencing.

Postnatal month	Family	% Mean relative abundance (SD)	
		UNS	EVC
		*n* = 11	*n* = 7
4	*Bifidobacteriaceae*	59.8 (27.0)	81.9 (9.72)
	*Coriobacteriaceae*	1.87 (5.62)	0.248 (0.479)
	*Bacteroidaceae*	7.19 (9.96)	4.45 (5.05)
	*Prevotellaceae*	0.001 (0.004)	0 (0.001)
	*Enterococcaceae*	0.476 (0.438)	1.02 (1.05)
	*Lactobacillaceae*	0.532 (0.711)	0.061 (0.159)
	*Clostridiaceae*	2.12 (5.77)	1.30 (1.99)
	*Lachnospiraceae*	8.67 (9.68)	1.81 (3.21)
	*Streptococcaceae*	1.19 (1.32)	2.41 (2.53)
	*Ruminococcaceae*	0.111 (0.257)	0.008 (0.009)
	*Veillonellaceae*	2.01 (3.50)	0.251 (0.340)
	*Erysipelotrichaceae*	3.72 (5.73)	0.161 (0.423)
	*Enterobacteriaceae*	8.31 (8.96)	4.77 (4.64)
	Other bacteria	4.03 (4.02)	1.62 (1.04)

		*n* = 16	*n* = 13
6	*Bifidobacteriaceae*	48.8 (26.9)	73.4 (16.2)**
	*Coriobacteriaceae*	1.68 (4.05)	0.232 (0.368)
	*Bacteroidaceae*	10.8 (13.7)	5.51 (6.65)
	*Prevotellaceae*	0.387 (1.52)	0.002 (0.003)
	*Enterococcaceae*	0.494 (0.759)	1.01 (0.867)
	*Lactobacillaceae*	2.49 (8.49)	0.693 (1.51)
	*Clostridiaceae*	1.17 (1.59)	1.26 (2.13)
	*Lachnospiraceae*	9.98 (11.3)	3.42 (7.95)*
	*Streptococcaceae*	1.19 (1.41)	1.79 (2.76)
	*Ruminococcaceae*	0.342 (0.534)	0.066 (0.169)
	*Veillonellaceae*	5.65 (6.60)	3.18 (5.30)
	*Erysipelotrichaceae*	2.57 (3.41)	0.248 (0.699)
	*Enterobacteriaceae*	9.18 (8.56)	6.34 (6.97)
	Other bacteria	5.19 (8.88)	2.81 (2.84)

		*n* = 18	*n* = 16
8	*Bifidobacteriaceae*	38.2 (24.6)	61.2 (23.6)*
	*Coriobacteriaceae*	1.49 (4.78)	0.349 (0.552)
	*Bacteroidaceae*	17.4 (19.6)	8.60 (10.8)
	*Prevotellaceae*	0.965 (3.58)	0.004 (0.008)
	*Enterococcaceae*	0.678 (0.855)	0.903 (0.938)
	*Lactobacillaceae*	0.942 (1.60)	1.09 (1.57)
	*Clostridiaceae*	2.30 (2.66)	2.62 (3.41)
	*Lachnospiraceae*	14.7 (15.3)	7.39 (13.2)*
	*Streptococcaceae*	1.00 (1.58)	1.20 (2.44)
	*Ruminococcaceae*	1.19 (2.32)	0.920 (3.00)
	*Veillonellaceae*	6.88 (4.24)	4.05 (4.27)
	*Erysipelotrichaceae*	2.07 (2.87)	0.872 (1.75)
	*Enterobacteriaceae*	7.94 (8.37)	7.21 (6.28)
	Other bacteria	4.25 (5.90)	3.64 (5.64)

		*n* = 19	*n* = 23
10	*Bifidobacteriaceae*	31.3 (20.7)	48.2 (24.7)*
	*Coriobacteriaceae*	1.39 (2.61)	0.733 (0.881)
	*Bacteroidaceae*	15.2 (17.2)	11.4 (12.5)
	*Prevotellaceae*	2.06 (5.88)	0.216 (0.744)
	*Enterococcaceae*	0.483 (0.540)	3.44 (13.1)
	*Lactobacillaceae*	1.11 (1.95)	0.656 (1.44)
	*Clostridiaceae*	1.53 (1.65)	1.62 (1.94)
	*Lachnospiraceae*	19.5 (15.4)	10.9 (13.5)*
	*Streptococcaceae*	0.776 (1.05)	1.09 (1.13)
	*Ruminococcaceae*	3.75 (8.52)	2.91 (5.50)
	*Veillonellaceae*	7.49 (7.99)	5.72 (6.28)
	*Erysipelotrichaceae*	2.69 (4.17)	0.699 (0.685)
	*Enterobacteriaceae*	6.76 (8.24)	4.58 (4.83)
	Other bacteria	5.90 (8.97)	7.80 (9.55)

		*n* = 21	*n* = 26
12	*Bifidobacteriaceae*	15.4 (14.0)	33.1 (22.4)**
	*Coriobacteriaceae*	0.930 (2.06)	0.779 (1.05)
	*Bacteroidaceae*	28.2 (19.1)	11.8 (11.1)**
	*Prevotellaceae*	2.18 (8.73)	2.16 (7.88)
	*Enterococcaceae*	0.169 (0.479)	0.657 (1.20)
	*Lactobacillaceae*	0.405 (1.17)	0.960 (2.04)
	*Clostridiaceae*	0.951 (1.12)	1.38 (1.31)
	*Lachnospiraceae*	24.6 (11.5)	17.4 (12.2)*
	*Streptococcaceae*	1.64 (1.99)	5.76 (14.7)
	*Ruminococcaceae*	10.2 (10.1)	7.49 (9.06)
	*Veillonellaceae*	5.55 (9.60)	7.15 (8.07)
	*Erysipelotrichaceae*	1.13 (1.31)	0.786 (1.08)
	*Enterobacteriaceae*	3.34 (5.29)	3.49 (4.40)
	Other bacteria	5.33 (4.46)	7.09 (5.92)

*P < 0.05.

**P < 0.01 for differences between treatments groups.

To investigate if supplementation with *B. infantis* resulted in differences in gut-related symptoms, mothers were asked how often infants experienced symptoms (never = 0, sometimes = 1, often = 2, very often = 3, unsure = 4, and refuse = 5, whereby unsure and refuse responses were excluded from the statistical analysis) and to rate the severity of these symptoms. The mean frequencies for gastrointestinal symptoms were not statistically significant between treatments at any study time point. Reported severity for infant constipation was 83% higher in the UNS vs. EVC group (*P* < 0.001); however, neither value was considered severe (Supplementary Table S2). The frequency for illnesses, sick doctor visits, hospitalizations, ear infections, respiratory tract infections, other infections, thrush, allergy, wheezing, asthma, eczema, and other conditions were not significantly different between treatments across time (Supplementary Table S3). There was also no difference in reported use of antibiotics, antigas medication, gripe water, probiotics with or without *B. infantis*, prescribed medications, or over-the-counter medications (Supplementary Table S4).

### Diversity analysis

Rarefaction curves were computed to assess differences in alpha-diversity composition as measured by the Shannon diversity index based on treatment status. No statistical difference was observed between groups (Supplementary Fig. 3a). Beta-diversity analysis was performed using UniFrac distances and the effect size of probiotic feeding was calculated, resulting in a significant (*P* = 0.001; adonis) although weak effect size (*R*^2^ = 0.05%; adonis) (Supplementary Fig. 3b).

### Follow-up #2

Of the 68 mothers enrolled in the parent IMPRINT Study, 51 mothers enrolled in the follow-up #2 study. Of these participants, *n* = 19 in the UNS and *n* = 17 in the EVC group completed the 18-month health questionnaire and *n* = 21 in the UNS and *n* = 20 in the EVC group completed the 24-month health questionnaire. There were no treatment differences in the number of children who experienced or were diagnosed with any common infant conditions or experiences (Supplementary Table S5). There were no significant differences in the mean number of experiences or diagnoses of common infant conditions (Supplementary Table S6).

## Discussion

The dominance of fecal *Bifidobacterium* and, specifically, *B. infantis* in the gut of breastfed infants has declined in recent decades in resource-rich nations, resulting in an increase in potential gut pathogens and immune dysfunction.^10,25–29^ Probiotic supplementation with *B. infantis* EVC001 in 7-day-old breastfed infants for 21 consecutive days resulted in a 7-log increase in fecal *B. infantis*, an increase in fecal *Bifidobacteriaceae* by 79%, a decrease in enteropathogens by 80%, an increase in fecal lactate and acetate by 2-fold, a decrease in fecal pH by 1-log,^19^ a decrease in antibiotic resistance genes, a sign of reduced enteropathogens known to harbor these genes,^30^ a reduction in mucin degradation,^31^ and reduced enteric inflammatory markers by several-fold^27^ during and 1 month post supplementation. These data demonstrate that the combination of breast milk and *B. infantis* EVC001 successfully restores the gut microbiome and biochemistry to historical norms observed a century ago.^9^

The infant gut microbiome is influenced by several maternal, dietary, and environmental factors, including delivery mode, feeding status (i.e., breast milk, infant formula, solid foods), and use of antibiotics. The current study showed that fecal *B. infantis* was 2.5–3.5 logs higher in infants in the EVC group compared with the UNS group at 6, 8, 10, and 12 months despite feeding status, and use of antibiotics or probiotics. The greatest difference in fecal *B. infantis* was observed in the earlier time points (6 and 8 months) when breast milk was the most abundant food source. The smallest difference in fecal *B. infantis* was observed at 12 months when infants’ diets were much more diverse and breast milk was less abundant. After excluding infants with confounding variables that impact the gut microbiome, such as infant formula, antibiotics, and probiotics, fecal *B. infantis* was 3.6–5.2 logs higher in infants in the EVC group compared with the UNS group at 6, 8, 10, and 12 months. We were unable to determine if probiotic intake during the 1-year follow-up period independently influenced fecal *B. infantis* abundance because six of the nine infants who consumed probiotics also received antibiotics. Taken together, these data suggest that a lack of HMOs, the preferred carbon source for *B. infantis*, and the use of antibiotics impact fecal *B. infantis* levels.

When infants were grouped by feeding type and exposures (breast milk fed without intake of infant formula, antibiotics or probiotics, mixed fed with breast milk and infant formula without intake of antibiotics or probiotics, and intake of antibiotics (all feeding types and probiotics), we found that fecal *B. infantis* was significantly higher in infants in the breast milk-fed group who were supplemented with *B. infantis* EVC001 compared with the UNS group. These findings further support the observation that breast milk is critical in supporting the colonization of *B. infantis*.

The UNS group had significantly higher *Lachnospiraceae*, including the genera, *Ruminococcus*, and *Blautia*, *Bacteroidaceae*, and lower *Bifidobacteriaceae* levels compared with the EVC group. These taxa differ in their preferences for carbohydrate substrates, metabolism of their preferred substrates into end products, and their consequent biochemical effects in the gut and on infant health. For example, members of the family *Lachnospiraceae* consist of spore-forming, anerobic bacteria that ferment complex plant polysaccharides into short-chain fatty acids (SCFAs), such as acetate, butyrate, and propionate.^32^ While gut microbes that produce SCFAs that lower luminal pH are considered beneficial, health outcomes associated with this family are mixed and likely vary with the genus or species and with host factors (e.g., infant vs. adult). For example, some members of this family that are commonly found in the human gut microbiome have been associated with a number of adverse health outcomes in adults (e.g., bloating, irritable bowel disease, metabolic disorders).^33–35^ In a prospective cohort study, the abundance of the family *Lachnospiraceae* at 3–4 months was higher in the gut of formula-fed infants compared to breastfed infants in a dose-dependent manner and associated with an 89% increase in the risk of overweight by 12 months.^36^ Emerging evidence suggests that the species, *Ruminococcus gnavus*, which belongs to the family *Lachnospiraceae*,^37^ may play a key role in allergy and immune development in infants^38^ and inflammation in the gut of adult patients with Crohn’s disease.^39^

In this study, we also found higher levels of the family *Bacteroidaceae* in the UNS group at 12 months postnatal. *Bacteroidaceae* is a family of Gram-negative, obligate anaerobic, nonsporulating bacilli that is commonly found in the healthy human adult colon. While most members of this family are considered commensals, some species, such as *Bacteroides fragilis*, include pathogenic strains.^40^ In addition, members of the *Bacteroidaceae* family contain an expanded set of genes encoded in polysaccharide utilization loci, allowing for the consumption of both dietary polysaccharides, as well as host-derived glycans.^41^ Specifically, *Bacteroides thetaiotaomicron* and *B. fragilis*, common members of the neonate gut, utilize a large set of mucin degradation polysaccharide utilization loci to catabolize HMOs.^42^ Previous studies in gnotobiotic mice have shown that downstream products derived from *Bacteroides*-driven HMO catabolism confer a growth advantage to potentially pathogenic *Enterobacteriaceae*, specifically *Escherichia coli*. This cross-feeding event was found to drive the *E. coli* bloom in a dextran sodium sulfate-induced colitis mouse model, thereby compounding the inflammatory response.^43^ On the other hand, the subspecies of *B. longum*, *B. infantis*, and specific strains of *B. infantis*^5^ such as EVC001 preferentially consume HMOs, which are fermented into acetate and lactate via the “bifid shunt.”^4,5^ These end products maintain a lower pH of the intestinal milieu, supporting the transport of these compounds into the intestinal epithelium for use by the host^6^ and creating an undesirable environment for potential pathogens.^7^ Acetate also blocks the infiltration of toxic molecules produced by pathogenic bacteria by enhancing intestinal barrier function and inhibiting proinflammatory and apoptotic responses.^8^ The clinical importance of infant fecal pH has been highlighted recently as a risk indicator for childhood stunting,^44^ and is also reflected in the updated reference range for infants provided by national diagnostic labs. The gut of infants enriched with the genus *Bifidobacterium* and low levels of potential pathogens decreases the risk of autoimmune diseases,^15,16^ supporting that supplementation with *B. infantis* EVC001 in early life may help protect infants from developing autoimmune diseases. Alpha-diversity was not different between groups; however, we found significant yet weak differences in beta-diversity between the two groups, suggesting that only a few OTUs were contributing to the overall beta-diversity in response to treatment status.

MaAsLin2 modeling also discovered that EVC supplementation was positively correlated with *Enterococcaceae* and *Enterococcus*. Confirmation of these data with statistical analyses at each time point found that *Enterococcaceae* was not different; however, *Enterococcus* was significantly higher by 0.5% in the EVC group compared with UNS at 6 months postnatal. Species that belong to the genus *Enterococcus* exert a range of functions in the gut as commensals to nosocomial pathogens that possess antibiotic resistance genes.^45^ In this study, *Enterococcus* represented a mean of 1% of the gut microbiome across both treatments and all time points, yet the variation was high ranging from 0.16% to 3.4% of the gut microbiome. For example, this genus represented 63% of the gut microbiome in one infant in the EVC group after the intake of antibiotics, but was reduced to 0% in this same infant 2 months later. We have previously reported that EVC supplementation reduced antibiotic resistance genes^30^ and that this taxon was not associated with enteric inflammation.^27^

In the follow-up #1 study, there was no difference in the frequency of illnesses, doctor visits, hospitalization, or health conditions between the EVC and UNS groups. Compared with the EVC group, participants in the UNS group reported a significantly higher score for the severity of their infants’ constipation (2.9 vs. 1.2), yet this value is not considered moderately or highly severe. In the follow-up #2 study, there were no differences between the EVC and UNS groups for the number of infants who were diagnosed with or experienced any common health conditions. While several larger studies have reported that probiotics can influence health conditions such as eczema,^46,47^ it is likely that the sample sizes in both follow-up #1 and follow-up #2 (*n* = 48 and *n* = 51, respectively) were too small to detect any significant differences in health outcomes or differences that may arise later in life.

One limitation of this study is that primers specific to the full genomic sequence for EVC001 were not used in this study. Based on the literature, *B. infantis* is an uncommon bifidobacterial subspecies found in infants who reside in Northern California.^19,25^ In the parent study published in Frese et al.,^19^ fecal *B. infantis* was on average 8 logs higher in infants supplemented with *B. infantis* EVC001 compared with UNS infants. Thus, we are confident that the several-fold difference in fecal *B. infantis* found in infants during the follow-up period is due to supplementation with EVC001 and not a random effect. Another limitation is that following completion of the parent study, factors that have confounding effects on the gut microbiome were not controlled. Although there were no significant differences in the number of infants among the different subgroups: breast milk; breast milk and infant formula; infant formula without breast milk; solid foods, or used antibiotics, probiotics, or were enrollees in daycare at any time point, given the small number in each subgroup it is possible that some of these factors had an impact on the gut microbiome. Second, different individuals participated in follow-up #1 and follow-up #2, limiting our ability to make direct comparisons between the gut microbiome results of follow-up #1 and the health outcomes measured in follow-up #2. Although follow-up #1 and #2 are independent of one another, both sets of participants stemmed from the parent study allowing us to make direct comparisons between treatment groups. Third, for both follow-up #1 and #2, not every participant provided a stool sample and questionnaire at every time point. As such, it was not possible to use paired data to compare the gut microbiome and health outcomes across time and, therefore, our statistical analyses were limited to the treatment group comparisons at each time point. Lastly, the parent study was originally designed to determine differences in the gut microbiome composition and fecal biochemistry at 1 month post-*B. infantis* EVC001 feeding and was not designed or powered to identify differences in health outcomes between treatment groups. Neither follow-up #1 nor #2 were designed or powered to detect differences in health outcomes between treatment groups. For example, previous longitudinal studies that have investigated the relationships between the early infant gut microbiome and atopic wheezing, and asthma have included both control and at-risk groups with sample sizes between 100 and 300 infants.^48,49^

Long-term colonization of a probiotic after cessation of its consumption has not been previously been demonstrated. These findings support the importance of matching a specific microorganism with a carbohydrate source that it selectively consumes thereby providing an open ecological niche for the microbe to occupy. We found that feeding breastfed infants a specific strain of *B. infantis* (EVC001) that efficiently utilizes all HMO structures in human milk for a brief period resulted in sustained colonization 1 year post supplementation. The gut microbiome in early infancy plays a critical role in immune system development and metabolic programming that has lifelong health impacts. Changes in the composition of the gut microbiome with lower protective microbes and higher potential pathogens associated with a Western lifestyle appear to increase the risks of developing allergic, inflammatory, and autoimmune diseases. Based on our findings, large clinical trials are warranted to determine whether *B. infantis* EVC001 supplementation early in life prevents the development of these diseases in child through adulthood.

## Supplementary information

Supplementary Figure 1

Supplementary Figure 2

Supplementary Figure 3

Supplementary Table 1
