# Supplementary material for: Early probiotic supplementation with B. infantis in breastfed infants leads to persistent colonization at 1 year
Source: Pediatr Res. 2021 Mar 24;91(3):627–36. doi: 10.1038/s41390-020-01350-0 (PMC8460680; doi:10.1038/s41390-020-01350-0)
Supplement: Supplementary file 1 — Supplementary Figure 1 [file 41390_2020_1350_MOESM1_ESM.pdf]

UCDAVIS FOODS FOR HEALTH INSTITUTE

Subject ID: __________

Health & Diet Questionnaire

Please answer the following questions regarding the diet and health of your baby who is enrolled in the Follow-up to IMPRINT Study. We also ask that you answer questions about your health and diet. Please mark your answers, and answer any follow-up questions as appropriate. We will not share your answers with anyone. If you prefer not to answer a question, you may check "refuse". If you are not sure, please write "Unsure" where appropriate.

TODAY'S DATE: _________________

Study Personnel Section:

Baby's DOB: _____________

IMPRINT completion date:__________

Actual Day of Life: _______ **Initials:** _______

Questions about your baby's Health

Questions	Answers
**1.** Has your baby shown any signs of COLIC **since completing the IMPRINT study**?<br><br>*[A colicky baby is defined as one that cries for more than 3 hours per day, for at least 3 days per week for at least one week]*<br><br>*[Mark only one answer]*	☐ Yes<br>☐ No ***[skip to question 4]***<br>☐ Unsure ***[skip to question 4]***<br>☐ Refuse ***[skip to question 4]***
**2.** Did your baby receive treatment for colic?<br><br>*[If you do not remember the treatment, write "unsure"]*	☐ Yes, what was the treatment(s)?: ________________________<br>☐ No<br>☐ Unsure<br>☐ Refuse
**3.** As of today, has the COLIC been resolved?	☐ Yes, when was it resolved: ______________(MM-YYYY)<br>☐ Yes, I don't remember when it was resolved<br>☐ No<br>☐ Unsure if it is resolved<br>☐ Refuse

Subject ID: __________

Health & Diet Questionnaire

**4.** How often did your baby experience the following symptoms **<u>since completing the IMPRINT study</u>**?<br><br>*[Mark only one for each symptom]*	

Subject ID: ___________

Health & Diet Questionnaire

	Never	Sometimes	Often	Very Often	Unsure	Refuse
Blood in stool	☐	☐	☐	☐	☐	☐
Burping or Belching	☐	☐	☐	☐	☐	☐
Constipation	☐	☐	☐	☐	☐	☐
Diarrhea	☐	☐	☐	☐	☐	☐
Discomfort in passing stool or gas	☐	☐	☐	☐	☐	☐
Eczema	☐	☐	☐	☐	☐	☐
Fatigue	☐	☐	☐	☐	☐	☐
Flatulence (farting)	☐	☐	☐	☐	☐	☐
	**Never**	**Sometimes**	**Often**	**Very Often**	**Unsure**	**Refuse**

Subject ID: ___________

Health & Diet Questionnaire

	Fever	☐	☐	☐	☐	☐	☐
	Foul-smelling flatulence	☐	☐	☐	☐	☐	☐
	Fussing without crying	☐	☐	☐	☐	☐	☐
	Irritability	☐	☐	☐	☐	☐	☐
	Rash	☐	☐	☐	☐	☐	☐
	Stuffy Nose	☐	☐	☐	☐	☐	☐
	Upset after spit-ups	☐	☐	☐	☐	☐	☐

Subject ID: ___________

Health & Diet Questionnaire

5. On a scale from 0-10, please rate the severity of common symptoms your baby may have experienced **since completing the IMPRINT study.**

[0 = least severe and 10 = Most severe]

[If your baby did not experience any symptoms, please mark "none"]

[If you are unsure if your baby experienced any symptoms, please mark "unsure"]

	None	1	2	3	4	5	6	7	8	9	10	Unsure	Refuse
Blood in stool	☐	☐	☐	☐	☐	☐	☐	☐	☐	☐	☐	☐	☐
Burping or Belching	☐	☐	☐	☐	☐	☐	☐	☐	☐	☐	☐	☐	☐
Constipation	☐	☐	☐	☐	☐	☐	☐	☐	☐	☐	☐	☐	☐
Diarrhea	☐	☐	☐	☐	☐	☐	☐	☐	☐	☐	☐	☐	☐
Discomfort in passing stool or gas	☐	☐	☐	☐	☐	☐	☐	☐	☐	☐	☐	☐	☐
Eczema	☐	☐	☐	☐	☐	☐	☐	☐	☐	☐	☐	☐	☐
Fatigue	☐	☐	☐	☐	☐	☐	☐	☐	☐	☐	☐	☐	☐
Flatulence (farting)	☐	☐	☐	☐	☐	☐	☐	☐	☐	☐	☐	☐	☐
Fever	☐	☐	☐	☐	☐	☐	☐	☐	☐	☐	☐	☐	☐

Health & Diet Questionnaire

	None	1	2	3	4	5	6	7	8	9	10	Unsure	Refuse
Foul-smelling flatulence	☐	☐	☐	☐	☐	☐	☐	☐	☐	☐	☐	☐	☐
Fussing without crying	☐	☐	☐	☐	☐	☐	☐	☐	☐	☐	☐	☐	☐
Irritability	☐	☐	☐	☐	☐	☐	☐	☐	☐	☐	☐	☐	☐
Rash	☐	☐	☐	☐	☐	☐	☐	☐	☐	☐	☐	☐	☐
Stuffy Nose	☐	☐	☐	☐	☐	☐	☐	☐	☐	☐	☐	☐	☐
Upset after spit-ups	☐	☐	☐	☐	☐	☐	☐	☐	☐	☐	☐	☐	☐

**6.** Has your baby been ill **since completing the IMPRINT study**?<br><br>*[Examples for illnesses include: cold, flu, ear infection, etc.]*<br><br>*[Mark only one answer]*	☐ Yes (please describe):____<br>____<br>☐ No<br>☐ Unsure<br>☐ Refuse
**7.** Did your baby have any SICK DOCTOR VISITS **since completing the IMPRINT study**?<br><br>*[A sick-child doctor visit is an appointment the parent makes when the baby is not feeling well]*<br><br>*[Mark only one answer]*	☐ Yes<br>☐ No ***[skip to question 9]***<br>☐ Unsure ***[skip to question 9]***<br>☐ Refuse ***[skip to question 9]***

Health & Diet Questionnaire

8. How many SICK DOCTOR VISITS did your baby have **<u>since completing the IMPRINT study</u>**, and what were the dates and reasons for these visits?

[If you are unable to find the exact date in your records, please include the month and year. If unsure, write unsure]

Sick Doctor Visits Since Completing IMPRINT:______

Date of Visit (MM/DD/YYYY)	Reason for Sick Doctor Visit

9. Was your baby hospitalized **<u>since completing the IMPRINT study</u>**?

☐ Yes

☐ No ***[skip to question 11]***

☐ Unsure ***[skip to question 11]***

☐ Refuse ***[skip to question 11]***

10. What were the reason(s) for and date(s) of hospitalizations?

[If you are unable to find the exact date in your records, please include the month and year. If unsure, write unsure]

**Date of Hospitilization (MM/DD/YYYY)**	**Reason for Hospitilization**

Subject ID: __________

Health & Diet Questionnaire

11. Was your baby diagnosed with any of the listed health conditions by a healthcare professional **<u>since completing the IMPRINT study</u>** and when?

[Mark all that apply]

[If your baby was not diagnosed with a condition, mark "not diagnosed"]

[If you are unable find the exact date in your records, please include the month and year. If unsure, write unsure]

Diagnosed Conditions	Not Diagnosed	Diagnosed	Date of Diagnosis (MM/DD/YYYY)
Ear infection	☐	☐	
Respiratory tract infection	☐	☐	
Thrush (yeast infection in the mouth)	☐	☐	
Yeast infection such as a diaper rash	☐	☐	
Other infection(s) (describe):____________ ______________________ ______________________ ______________________	☐	☐	
Allergy	☐	☐	
Asthma	☐	☐	
Eczema	☐	☐	
Wheezing	☐	☐	
Other conditions (describe):____________ ______________________ ______________________	☐	☐	

Health & Diet Questionnaire

12. Did your baby take any oral or IV antibiotics at any time **since completing the IMPRINT study**?

[Mark only one answer]

- ☐ Yes
- ☐ No ***[skip to question 14]***
- ☐ Unsure ***[skip to question 14]***
- ☐ Refuse ***[skip to question 14]***

13. Which oral/IV antibiotics did your baby take, what were the number of days your baby took the oral antibiotic, the start and end dates, and reasons he/she took them?

[If you are unable find the exact date in your records, please include the month and year. If unsure, write unsure]

Oral/IV antibiotic name	Number of days oral/IV antibiotic was taken	Reason for oral/IV antibiotic	Start Date	End Date [Or still taking]
Ex: dicloxacillin	*7*	*To treat an ear infection*	*4/10/16*	*4/16/16*

14. Did your baby take any of the following medications, supplements or vitamins at any time **since completing the IMPRINT study**?

[Mark all that apply]

[Mark "none" if your baby did not consume any of these items]

	Yes	No	Unsure	Refuse
Probiotics	☐	☐	☐	☐
Anti-gas drops (such as Simethicone, Mylicon, etc)	☐	☐	☐	☐
Gripe water	☐	☐	☐	☐
Fish oil or long chain omega 3 fatty acids (EPA or DHA)	☐	☐	☐	☐
Vitamin D drops	☐	☐	☐	☐

Subject ID: ___________

Health & Diet Questionnaire

	Yes	No	Unsure	Refuse
Other vitamin drops	☐	☐	☐	☐
Over-the-counter medications	☐	☐	☐	☐
Prescribed medications	☐	☐	☐	☐

Subject ID: __________

Health & Diet Questionnaire

15. Please list the medications, supplements or vitamins marked in Question 14, type, brand name, product name, strength, doses per day, number of days per week your baby took the dose, start and end dates, and reasons he/she took them.

[If you are unable find the exact date in your records, please include the month and year. If unsure, write unsure]

Type of product	Brand and Product Name	Strength	How many doses did your baby take per day?	How many days per week did your baby take this dose?	Start Date	End Date (or still taking it?)	Reason for Medication
Example: *Ibuprofen*	*Motrin*	*1.25ml*	*1*	*1*	*5/15/2015*	*5/15/2015*	*Fever*
Example: *Probiotic*	*Udo's, Choice Infant's Blend Probiotic*	*¼ teaspoon*	*1*	*5*	*5/10/2015*	*Still taking*	*Gassy and colicky*

16. Did your baby have a **WELL-CHILD DOCTOR VISIT since completing the IMPRINT study**?

[A well-child doctor visit is a routine appointment that gives the doctor a chance to look at your baby's overall health]

- ☐ Yes, Date of visit (MM-DD-YYYY):__________________
- ☐ No ***[skip to question 20]***
- ☐ Unsure ***[skip to question 20]***
- ☐ Refuse ***[skip to question 20]***

17. Was your baby's weight measured during this **WELL-CHILD DOCTOR VISIT?**

[Mark only one answer]

- ☐ Yes, my baby's weight was (pounds and ounces):____________
- ☐ Yes, but I am unsure of what my baby's weight was
- ☐ No, my baby's weight was not measured at this visit
- ☐ Unsure
- ☐ Refuse

Health & Diet Questionnaire

**18.** Was your baby's height measured during this **WELL-CHILD DOCTOR VISIT?**<br><br>*[Mark only one answer]*	☐ Yes, my baby's height was (inches): ____________<br>☐ Yes, but I am unsure of what my baby's height was<br>☐ No, my baby's height was not measured during this visit<br>☐ Unsure<br>☐ Refuse
**19.** Were any medical problems reviewed by your baby's **WELL-CHILD DOCTOR VISIT**?<br><br>*[Mark only one answer]*	☐ Yes (please describe):______________________________________<br>______________________________________<br>______________________________________<br>☐ No<br>☐ Unsure<br>☐ Refuse
**20.** Did your baby have any vaccinations **since completing the IMPRINT study** and when?<br><br>*[Mark all that apply]*<br><br>*[Mark "none" if your baby did not receive any vaccinations]*<br><br>*[If you are unable find the exact date in your records, please include the month and year. If unsure, write unsure]*	**Which vaccinations did your baby receive?** (see table below)

Vacccinations	Did Not Receive	Received	Date Vaccination Was Given (MM/DD/YYYY)
Diphtheria / Tetanus / Pertussis (DTaP)	☐	☐	
Haemophilus influenzae type b (Hib)	☐	☐	
Hepatitis A (Hep A)	☐	☐	
Hepatitis B (Hep B)	☐	☐	
Influenza (Flu)	☐	☐	

Subject ID: ___________

Health & Diet Questionnaire

Vacccinations	Did Not Receive	Received	Date Vaccination Was Given (MM/DD/YYYY)
Measles, mumps, rubella (MMR)	☐	☐	
Pneumococcal (PCV)	☐	☐	
Polio	☐	☐	
Rotavirus (RV)	☐	☐	
Varicella (chickenpox)	☐	☐	
Other (list):	☐	☐	

Subject ID: __________

Health & Diet Questionnaire

Questions about your baby's diet

Questions	Answers
**21.** Please mark how your baby has been fed **since completing the IMPRINT study**?<br><br>*[Mark all that apply]*<br>*[If your baby has not reached the age of a listed month range, please mark "Age does not Apply"]*	see table below
**22.** How old was your baby when he/she **first** consumed infant formula?<br><br>*[Mark only one answer]*	☐ My baby has not consumed any infant formula. ***[skip to question 26]***<br><br>☐ My Baby was: __________day or weeks **[circle]** old when she/he first consumed infant formula.
**23.** Is your baby still consuming any infant formula?	☐ Yes ***[skip to question 25]***<br><br>☐ No
**24.** How old was your baby when he/she **last** consumed infant formula?	☐ My Baby was: __________days or weeks **[circle]** old when she/he last consumed infant formula.

**Age (Month)**	**Breast Milk**	**Formula**	**Solids**
2-3 months	☐ Yes<br>☐ No<br>☐ Unsure<br>☐ Age does not apply	☐ Yes<br>☐ No<br>☐ Unsure<br>☐ Age does not apply	☐ Yes<br>☐ No<br>☐ Unsure<br>☐ Age does not apply
3-4 months	☐ Yes<br>☐ No<br>☐ Unsure<br>☐ Age does not apply	☐ Yes<br>☐ No<br>☐ Unsure<br>☐ Age does not apply	☐ Yes<br>☐ No<br>☐ Unsure<br>☐ Age does not apply
4-5 months	☐ Yes<br>☐ No<br>☐ Unsure<br>☐ Age does not apply	☐ Yes<br>☐ No<br>☐ Unsure<br>☐ Age does not apply	☐ Yes<br>☐ No<br>☐ Unsure<br>☐ Age does not apply

Subject ID: __________

Health & Diet Questionnaire

25. Mark approximately how much formula your baby ate at each time period.

[Mark only one answer for each time period]

[For this question, we are not asking about the amount of infant formula in each bottle]

[If your baby has not reached the age of a listed month range, mark "Age does not apply"]

Age (Month)	No formula during this time period	1-2 bottles of formula per day	3-5 bottles of formula per day	6-8 bottles of formula per day	> 8 bottles of formula per day	Unsure of how much formula	Age Does not apply
2-3 months	☐	☐	☐	☐	☐	☐	☐
3-4 months	☐	☐	☐	☐	☐	☐	☐
4-5 months	☐	☐	☐	☐	☐	☐	☐

26. Did your baby consume **any** solid food **since completing the IMPRINT study**?

[Solid foods are any food or beverages other than breastmilk, infant formula, or water]

[Mark only one answer]

☐ My baby has not yet consumed any solid food since completing the IMPRINT study. ***[skip to question 35]***

☐ Yes, my baby has consumed solids since completing the IMPRINT study.

Health & Diet Questionnaire

27. Did your baby eat any **<u>grains or cereals</u>** in the **<u>past week</u>** and if so how much did he/she eat?

[For food mixtures, mark "mixtures" and list the foods in the mixture and list the total amount of the mixture your baby ate]

[Mark all that apply]

Grains or Cereals	No	Yes	Amount (total number of tablespoons your baby ate this past week)	Unsure	Refuse
Rice	☐	☐		☐	☐
Oats or oatmeal	☐	☐		☐	☐
Barley	☐	☐		☐	☐
Spelt	☐	☐		☐	☐
Other: ________ ________	☐	☐		☐	☐
Mixtures: ________ ________	☐	☐		☐	☐

Health & Diet Questionnaire

28. Did your baby eat any **starches** in the **past week** and if so how much did he/she eat?

[For food mixtures, mark "mixtures" and list the foods in the mixture and list the total amount of the mixture your baby ate]

[Mark all that apply]

Starches	No	Yes	Amount (total number of tablespoons your baby ate this past week)	Unsure	Refuse
Baby biscuits	☐	☐		☐	☐
Puff snacks without any added sugar	☐	☐		☐	☐
Puff snacks with added sugar	☐	☐		☐	☐
Pasta, wheat	☐	☐		☐	☐
Pasta, rice	☐	☐		☐	☐
Bread	☐	☐		☐	☐
Tortilla	☐	☐		☐	☐
Other: _____________	☐	☐		☐	☐
Mixtures: _____________ _____________	☐	☐		☐	☐

Health & Diet Questionnaire

29. Did your baby eat any **vegetables or legumes** in the **past week** and if so how much did he/she eat?

[For food mixtures, mark "mixtures" and list the foods in the mixture and list the total amount of the mixture your baby ate]

[Mark all that apply]

Vegetables or Legumes	No	Yes	Amount (total number of tablespoons your baby ate this past week)	Unsure	Refuse
Carrots	☐	☐		☐	☐
Peas	☐	☐		☐	☐
Sweet Potatoes	☐	☐		☐	☐
Beets	☐	☐		☐	☐
String beans	☐	☐		☐	☐
Squash	☐	☐		☐	☐
Butternut squash	☐	☐		☐	☐
Corn	☐	☐		☐	☐
Spinach	☐	☐		☐	☐
Potato	☐	☐		☐	☐

Health & Diet Questionnaire

Vegetables or Legumes	No	Yes	Amount (total number of tablespoons your baby ate this past week)	Unsure	Refuse
Avocado	☐	☐		☐	☐
Zucchini	☐	☐		☐	☐
Broccoli	☐	☐		☐	☐
Pumpkin	☐	☐		☐	☐
Lentils	☐	☐		☐	☐
Beans, black	☐	☐		☐	☐
Beans, pinto	☐	☐		☐	☐
Beans, fava	☐	☐		☐	☐
Beans, kidney	☐	☐		☐	☐
Beans, garbanzo	☐	☐		☐	☐

Health & Diet Questionnaire

Vegetables or Legumes	No	Yes	Amount (total number of tablespoons your baby ate this past week)	Unsure	Refuse
Kale	☐	☐		☐	☐
Other: ____________	☐	☐		☐	☐
Mixtures: ____________ ____________	☐	☐		☐	☐

30. Did your baby eat any **fruits** in the **past week** and if so how much did he/she eat?

[For food mixtures, mark "mixtures" and list the foods in the mixture and list the total amount of the mixture your baby ate]

[Mark all that apply]

Fruit	No	Yes	Amount (total number of tablespoons your baby ate this past week)	Unsure	Refuse
Apples	☐	☐		☐	☐
Bananas	☐	☐		☐	☐
Prunes	☐	☐		☐	☐
Plums	☐	☐		☐	☐
Apricots	☐	☐		☐	☐

Health & Diet Questionnaire

Fruit	No	Yes	Amount (total number of tablespoons your baby ate this past week)	Unsure	Refuse
Blueberries	☐	☐		☐	☐
Raspberries	☐	☐		☐	☐
Blackberries	☐	☐		☐	☐
Strawberries	☐	☐		☐	☐
Peaches	☐	☐		☐	☐
Pears	☐	☐		☐	☐
Mangos	☐	☐		☐	☐
Melon	☐	☐		☐	☐
Figs	☐	☐		☐	☐
Kiwi	☐	☐		☐	☐

Health & Diet Questionnaire

Fruit	No	Yes	Amount (total number of tablespoons your baby ate this past week)	Unsure	Refuse
Pineapple	☐	☐		☐	☐
Orange	☐	☐		☐	☐
Other: _____________	☐	☐		☐	☐
Mixtures: _____________ _____________	☐	☐		☐	☐

31. Did your baby eat any **meat** in the **past week** and if so how much did he/she eat?

[For food mixtures, mark "mixtures" and list the foods in the mixture and list the total amount of the mixture your baby ate]

[Mark all that apply]

Meat	No	Yes	Amount (total number of tablespoons your baby ate this past week)	Unsure	Refuse
Chicken	☐	☐		☐	☐
Turkey	☐	☐		☐	☐
Fish with scales	☐	☐		☐	☐
Shellfish	☐	☐		☐	☐

Health & Diet Questionnaire

Meat	No	Yes	Amount (total number of tablespoons your baby ate this past week)	Unsure	Refuse
Lamb	☐	☐		☐	☐
Pork	☐	☐		☐	☐
Beef	☐	☐		☐	☐
Other: ____________	☐	☐		☐	☐
Mixtures: ____________ ____________ ____________	☐	☐		☐	☐

Health & Diet Questionnaire

32. Did your baby eat any **eggs or dairy** in the **past week** and if so how much did he/she eat?

[For food mixtures, mark "mixtures" and list the foods in the mixture and list the total amount of the mixture your baby ate]

[one ounce of hard cheese or Mozzarella cheese are equal to the size of three dice]

[Mark all that apply]

Dairy	No	Yes	Amount (total number of tablespoons your baby ate this past week)	Unsure	Refuse
Eggs, whole	☐	☐		☐	☐
Eggs, yolk only	☐	☐		☐	☐
Eggs, white only	☐	☐		☐	☐
Yogurt	☐	☐		☐	☐
Milk, whole	☐	☐		☐	☐
Milk, 2%	☐	☐		☐	☐
Milk, 0% or 1%	☐	☐		☐	☐
Full fat cheese	☐	☐		☐	☐
Mozzarella cheese	☐	☐		☐	☐
Cottage cheese	☐	☐		☐	☐
Other: ____________	☐	☐		☐	☐
Mixtures: ____________ ____________	☐	☐		☐	☐

Health & Diet Questionnaire

33. Did your baby eat any **chunky blends of food combinations** in the **past week** and if so list the combinations and the amount he/she ate?

[For chunky blends we are asking about complex meals not listed above such as stews, soups, etc.]

☐ No, my baby has not consumed any chunky blends of food combinations in the past week.

☐ Yes, my baby has consumed chunky blends of food combinations in the past week and listed in the table below.

☐ Unsure

☐ Refuse

**Chunky blends of food combinations (please list)**	**Amount** **(total number of tablespoons your baby ate this past week)**

34. Did your baby drink any **beverages** other than breastmilk or infant formula in the **past week** and if so how much did she/he eat?

[Exclude water]

[For mixtures, mark "mixtures" and list the foods in the mixture and list the total amount of the mixture your baby drank]

[Mark all that apply]

**Beverages**	**No**	**Yes**	**Amount** **(total number of tablespoons your baby ate this past week)**	**Unsure**	**Refuse**
Fruit juice	☐	☐		☐	☐
Vegetable juice	☐	☐		☐	☐
Coconut water	☐	☐		☐	☐

Health & Diet Questionnaire

Beverages	No	Yes	Amount (total number of <u>tablespoons</u> your baby ate this past week)	Unsure	Refuse
Coconut milk	☐	☐		☐	☐
Soy milk	☐	☐		☐	☐
Almond milk	☐	☐		☐	☐
Other: ____________	☐	☐		☐	☐
Mixtures: ____________ ____________	☐	☐		☐	☐

Questions about your baby's <u>Environment</u>

Questions	Answers
**35.** On average, how many baths did your baby receive per week **<u>since completing the IMPRINT study</u>**?	**# of baths/week with water only:______**<br>**# of baths/week with soap and water:_____**<br>*[This includes any type of soap, including body gel and shampoo]:*<br>Please list the brand(s) of soap used.<br>______________________________<br>☐ Unsure<br>☐ Refuse

Health & Diet Questionnaire

**36.** If your baby's pacifier or toy fell on an indoor floor today, what would you **TYPICALLY** do before handing it back to your baby?<br><br>*[Mark only one answer]*	☐ I would wash it with soap and water<br>☐ I would rinse it with water<br>☐ I would wipe it off<br>☐ I would lick it off<br>☐ I would not do anything and hand it back to him/her<br>☐ Other: ______________________________
**37.** Did your baby attend any part-time or full time daycare programs **since completing the IMPRINT study?**<br><br>*[Includes formal and at-home daycares in which other babies are enrolled]*<br><br>*[Mark only one answer]*	☐ Yes<br>☐ No ***[skip to question 43]***<br>☐ Unsure ***[skip to question 43]***<br>☐ Refuse ***[skip to question 43]***
**38.** Approximately how old was your baby when he/she was first enrolled into daycare?	|___|___| <br>**Weeks old**
**39.** How many different daycares has your baby attended **since completing the IMPRINT study**?<br><br>*[Mark only one answer]*	☐ 1 daycare<br>☐ 2-3 daycares<br>☐ More than 3 daycares<br>☐ Unsure<br>☐ Refuse
**40.** Approximately, what is the most number of days per week that your baby spent at any daycare **since completing the IMPRINT study**?<br><br>*[Mark only one answer]*	☐ 1-2 days/week<br>☐ 2-3 days/week<br>☐ 3-5 days/week<br>☐ Unsure<br>☐ Refuse

Subject ID: __________

Health & Diet Questionnaire

**41.** Approximately what is the most number of hours per day your baby spent in any daycare **since completing the IMPRINT study**?<br><br>*[Mark only one answer]*	☐ 1-2 hours/day<br>☐ 2-4 hours/day<br>☐ 4-6 hours/day<br>☐ More than 6 hours/day<br>☐ Unsure<br>☐ Refuse
**42.** To your knowledge, were any other infants in any of your baby's daycare programs breastfed (at the breast or by bottle)?<br><br>*[Mark only one answer]*	☐ Yes<br>☐ No<br>☐ Unsure<br>☐ Refuse
**43.** Has anyone other than you nursed your baby at the breast **since completing the IMPRINT study?**<br><br>*[Mark only one answer]*	☐ Yes<br>☐ No ***[skip to question 45]***<br>☐ Unsure ***[skip to question 45]***<br>☐ Refuse ***[skip to question 45]***
**44.** Who other than you nursed your baby **since completing the IMPRINT study**?<br><br>*[Mark all that apply]*	☐ A friend<br>☐ A neighbor<br>☐ The baby's aunt<br>☐ The baby's sister<br>☐ The baby's grandmother<br>☐ The baby's nanny<br>☐ Other: (please specify) ______________________________________

If your baby has consumed breastmilk at the breast or by bottle since completing the IMPRINT study, continue to the next section.

If your baby has NOT consumed breastmilk since completing the IMPRINT study,
End of Questionnaire

Subject ID: __________

Health & Diet Questionnaire

Questions about YOU if your baby has consumed breastmilk at the breast or by bottle since completing the IMPRINT study

Questions	Answers
**45.** Have you experienced any illnesses **since completing the IMPRINT study**?<br><br>*[An illness is any episode of feeling unwell such as a cold, sinus infection, yeast infection, etc.]*<br><br>*[Mark only one answer]*	☐ Yes<br>☐ No ***[skip to question 47]***<br>☐ Unsure ***[skip to question 47]***<br>☐ Refuse ***[skip to question 47]***
**46.** What were the illnesses and when did you experience them?<br><br>*[An illness is any episode of feeling unwell such as a cold, sinus infection, yeast infection, etc.]*<br><br>*[If you are unable to find the exact date in your records, please include the month and year. If unsure, write unsure]*	(see table below)
**47.** Have you taken any oral or IV antibiotics **since completing the IMPRINT study**?<br><br>*[Mark only one answer]*	☐ Yes<br>☐ No ***[skip to question 49]***<br>☐ Unsure ***[skip to question 49]***<br>☐ Refuse ***[skip to question 49]***

**Illness**	**Date of illness (MM/DD/YYYY)**

Health & Diet Questionnaire

48. Which oral or IV antibiotics did you take, what were the number of days you took the antibiotic, the start and end dates, and reasons for taking them?

[If you are unable to find the exact date in your records, please include the month and year. If unsure, write unsure]

Oral/IV antibiotic name	Number of days oral/IV antibiotic was taken	Start Date	End Date (or are you still taking it?)	Reason for oral/IV antibiotic
Ex: dicloxacillin	*7*	*4/1/16*	*4/6/16*	*To treat mastitis*

49. Did you take any of the following vitamins or supplements **since completing the IMPRINT study**?

[Mark all that apply]

Vitamins/Supplements	Yes	No	Unsure	Refuse
Beta Carotene	☐	☐	☐	☐
B complexes (stress tabs)	☐	☐	☐	☐
Calcium	☐	☐	☐	☐
Co-enzyme Q10	☐	☐	☐	☐
Flaxseeds, flaxseed meal, flaxseed oil or chia seeds	☐	☐	☐	☐
Fish oil	☐	☐	☐	☐

Subject ID: ___________

Health & Diet Questionnaire

Vitamins/Supplements	Yes	No	Unsure	Refuse
Folic acid	☐	☐	☐	☐
Ginseng	☐	☐	☐	☐
Ginko biloba	☐	☐	☐	☐
Iron	☐	☐	☐	☐
Multivitamin with iron	☐	☐	☐	☐
Multivitamin without iron	☐	☐	☐	☐
Niacin	☐	☐	☐	☐
Prenatal vitamin with iron	☐	☐	☐	☐
Prenatal vitamin without iron	☐	☐	☐	☐
Probiotics	☐	☐	☐	☐
Selenium	☐	☐	☐	☐

Health & Diet Questionnaire

Vitamins/Supplements	Yes	No	Unsure	Refuse
St. John's Wort	☐	☐	☐	☐
Vitamin A/Retinol	☐	☐	☐	☐
Vitamin B1	☐	☐	☐	☐
Vitamin B6	☐	☐	☐	☐
Vitamin B12	☐	☐	☐	☐
Vitamin C	☐	☐	☐	☐
Vitamin D	☐	☐	☐	☐
Vitamin E	☐	☐	☐	☐
Vitamin K	☐	☐	☐	☐
Yogurt containing bifidobacteria	☐	☐	☐	☐
Zinc	☐	☐	☐	☐

Health & Diet Questionnaire

Vitamins/Supplements	Yes	No	Unsure	Refuse
Other (please list):	☐	☐	☐	☐

50. If you marked in Question 49 that you took **probiotics since completing the IMPRINT study**, please list all of the name brands and product names you used, amount consumed, number of days per week you took the probiotics, and the dates taken.

[If you did not mark that you took any probiotics in Question 49, check the box "I did not take any probiotics"]

[If you are unable to find the exact date in your records, please include the month and year. If unsure, write unsure]

Probiotic Brand and Product Name	Amount/d	How many days per week did you take the daily dose?	Start Date (MM/DD/YYYY)	End Date (MM/DD/YYYY)
Example: VSL #3	*2 capsules*	*5*	*1/15/2014*	*6/1/2014*

☐ I did not take probiotics since completing the IMPRINT study

51. If you marked in Question 49 that you took **fish oil since completing the IMPRINT study**, please list all of the name brands you used, dose per day, number of days per week you took the dose, and the dates taken.

[If you did not mark that you took any fish oil in Question 49, check the box "I did not take any fish oil"]

[If you are unable to find the exact date in your records, please include the month and year. If unsure, write unsure]

Fish Oil Brand Name	Product Name	Amount /day	How many days per week do you take the daily dose?	Start Date (MM/DD/YYYY)	End Date (MM/DD/YYYY)
Example: Nordic Naturals	*DHA Extra*	*2 capsules*	*7*	*1/15/2014*	*10/1/2014*

Subject ID: __________

Health & Diet Questionnaire

	<table><tr><th>Fish Oil Brand Name</th><th>Product Name</th><th>Amount /day</th><th>How many days per week do you take the daily dose?</th><th>Start Date (MM/DD/YYYY)</th><th>End Date (MM/DD/YYYY)</th></tr><tr><td></td><td></td><td></td><td></td><td></td><td></td></tr><tr><td></td><td></td><td></td><td></td><td></td><td></td></tr></table> ☐ I did not take any fish oil since completing the IMPRINT study
**52.** How many servings of scaly fish did you eat **since completing the IMPRINT study?** *[one 3 ounce serving of fish is the size of a deck of cards]*	**# of servings**: ________<br>☐ I did not eat any scaly fish since completing the IMPRINT study<br>☐ Unsure<br>☐ Refuse
**53.** How many servings of shellfish did you eat **since completing the IMPRINT study**? *[one 3 ounce serving of shellfish is the size of a deck of cards]*	**# of servings**: ________<br>☐ I did not eat shellfish since completing the IMPRINT study<br>☐ Unsure<br>☐ Refuse

End of the Questionnaire

Subject ID: __________

Health & Diet Questionnaire

Study Personnel Section

Instructions for weighing the baby on a portable Tanita infant scale:

1. Set up the scale: plug the power cord into an electrical outlet, remove the baby scale cushion and record the scale code here: ______

2. Ask a parent to remove clothes and the diaper from the baby.

3. Turn on the scale. Press the "grams/pounds & ounces" button to read weight in "grams".

4. Place a disposable liner on the scale and press "tare" to zero the scale.

5. Ask a parent to place the baby on the scale. Without using any pressure that would add to the weight of the baby, make sure he/she will not roll off the scale.

6. After the weight is stabilized, record the weight here: ___________ grams

7. To obtain the weight in pounds & ounces for the parent's interest, ask the parent to remove the baby, turn the scale off and then on and press the "grams/pounds & ounces" to read the weight in "pounds and ounces".

8. Ask a parent to remove the infant from the scale and diaper/dress as normal.

9. Dispose the scale liner in the trash before packing up the scale.
