# Supplementary material for: Early probiotic supplementation with B. infantis in breastfed infants leads to persistent colonization at 1 year
Source: Pediatr Res. 2021 Mar 24;91(3):627–36. doi: 10.1038/s41390-020-01350-0 (PMC8460680; doi:10.1038/s41390-020-01350-0)
Supplement: Supplementary file 2 — Supplementary Figure 2 [file 41390_2020_1350_MOESM2_ESM.pdf]

Questionnaire instructions

IMPRINT Follow-up #2

Health Questionnaire

Questionnaire Instructions

Purpose:

This questionnaire will ask about your and your child's health and diet. We hope to understand how your child's gut bacteria during the study is associated with his/her health today. We will not share your answers with anyone. This questionnaire will take you approximately 10-20 minutes to complete.

Instructions:

Please try and give your best answer to each question. Provide only one answer to each question unless instructed to “mark all that apply”. If you do not know or cannot remember an answer, you may mark “unsure”. If you prefer not to answer a question, you may mark “refuse”.

You may stop taking the online questionnaire and re-open it on any device using the link we sent to your email address. Once you begin the questionnaire, you will have one week to complete it. You may go back to previous questions using the back arrow on the bottom of the questionnaire, but you will not be able to move forward in the questionnaire until you provide an answer to every question. Once

you submit your completed questionnaire, you will not be able to go back and change your answers.

In this questionnaire, we will ask you about your baby's height and weight measured at his/her 18 month Well-Child doctor visit; please gather this information from his/her health records before starting this questionnaire. Only take this questionnaire after having this visit.

Questions about your baby's health Part 1

Questions about your child's health

Has your child had an 18 month **Well Child visit**?

○ Yes
○ No
○ Unsure

Please complete this survey after your child has had his/her 18 month **Well Child visit**.

When was your child's 18 month **Well Child visit**?

[Please approximate the date if you are unsure or select "unsure"]

	Month	Day	Year
Date			

When was your child's 18 month **Well Child visit**?

[Please approximate the date if you are unsure or select "unsure"]

	Month	Day	Year
Date			

What was his/her measured **height**?

	Feet	Inches
Height		

What was his/her measured **weight**?

	Pounds	Ounces
Weight		

Has your child EVER been **diagnosed** with **eczema** by a health care provider?

[Eczema shows up as patches of red skin. The skin is almost always itchy, dry, and rough and most often occurs on a child's cheeks and at the joints of their arms and legs]

- Yes
- No
- Unsure
- Refuse

How many new episodes of **eczema** has your child experienced in the past month?

[A new episode of eczema is the appearance of a new rash after a previous rash has completely disappeared]

How **severe** was your child's **worst eczema rash** in the past month?

[1 is least severe and 10 is most severe]

	Least severe										Most severe
	0	1	2	3	4	5	6	7	8	9	10
How severe?											

Has your child EVER been **diagnosed** with **asthma** by a health care provider?

- ○ Yes
- ○ No
- ○ Unsure
- ○ Refuse

Has your child EVER been **diagnosed** with **wheezing** by a health care provider?

- ○ Yes
- ○ No
- ○ Unsure
- ○ Refuse

Has your child EVER been **diagnosed** with **gastroesophogeal reflux disease (GERD)** by a health care provider?

[GERD is a chronic digestive disease that occurs when stomach acid or, occasionally, stomach content, flows back into your food pipe (esophagus). The backwash (reflux) irritates the lining of your esophagus and causes GERD]

- ○ Yes
- ○ No
- ○ Unsure
- ○ Refuse

Questions about your baby's health Part 2

Has your child EVER been **diagnosed** with **lactose intolerance** by a health care provider?

- ○ Yes
- ○ No
- ○ Unsure
- ○ Refuse

Has your child EVER been **diagnosed** with any **allergies** by a health care provider?

[We are asking about a diagnosis based on tests performed by a health care provider. This question is not asking about intolerances such as lactose intolerance]

- ○ Yes
- ○ No
- ○ Unsure
- ○ Refuse

Has your child EVER been **diagnosed** with any of the following specific **allergies**?

[We are asking about a diagnosis based on tests performed by a health care provider. This question is not asking about intolerances such as lactose intolerance]

	Yes	No	Unsure	Refuse
Animal dander allergy	○	○	○	○
Dust allergy	○	○	○	○
Food allergy	○	○	○	○
Insect sting allergy	○	○	○	○
Latex allergy	○	○	○	○
Medication allergy	○	○	○	○
Mold allergy	○	○	○	○
Pollen allergy	○	○	○	○
Other (please describe in the next question)	○	○	○	○

Which **animal dander allergy(s)** has your child EVER been **diagnosed** with?

____

Has your child EVER been **diagnosed** with any of the following **food allergies**?

	Yes	No	Unsure	Refuse
Eggs	○	○	○	○
Fish with fins (tuna, bass, salmon, shark, trout, etc.)	○	○	○	○

	Yes	No	Unsure	Refuse
Milk protein	○	○	○	○
Peanuts	○	○	○	○
Shellfish (clams, crab, scallops, shrimp, squid, etc.)	○	○	○	○
Soy	○	○	○	○
Tree nuts (walnuts, cashews, etc.)	○	○	○	○
Wheat	○	○	○	○
Other (please describe in the next question)	○	○	○	○

What **other food allergies** has your child EVER been **diagnosed** with?

____

What **medication allergy(s)** has your child EVER been **diagnosed** with?

____

What **other allergy(s)** has your child EVER been **diagnosed** with?

____

Have any of your child's **allergy diagnoses changed** since he/she was first diagnosed?

[For this question, we are asking about any allergies that your child outgrew]

○ Yes

○ No

○ Unsure

○ Refuse

Please describe how your child's allergy diagnoses have changed.

Has your child EVER **experienced** any **allergic reactions**?

[Think of allergic reactions to both foods such as nuts, fish, seafood, eggs, milk protein, wheat, etc., and to environmental antigens such as dust, pollen, mites, dander, grasses, etc. This question is not asking about intolerances such as lactose intolerance]

- ○ Yes
- ○ No
- ○ Unsure
- ○ Refuse

Has your child EVER **experienced** any of the following specific **allergic reactions**?

[Think of allergic reactions to both foods such as nuts, fish, seafood, eggs, milk protein, wheat, etc., and to environmental antigens such as dust, pollen, mites, dander, grasses, etc. This question is not asking about intolerances such as lactose intolerance]

	Yes	No	Unsure	Refuse
Animal dander	○	○	○	○
Dust	○	○	○	○
Food	○	○	○	○
Insect sting	○	○	○	○
Latex	○	○	○	○
Medication	○	○	○	○
Mold	○	○	○	○
Pollen	○	○	○	○
Other (please describe in the next question)	○	○	○	○

What **animal dander(s)** has EVER caused any **allergic reactions** in your child?

Have any of the following **foods** EVER caused any **allergic reactions** in your child?

	Yes	No	Unsure	Refuse
Eggs	○	○	○	○
Fish with fins (tuna, bass, salmon, shark, trout, etc.)	○	○	○	○
Milk protein	○	○	○	○
Peanuts	○	○	○	○
Shellfish (clams, crab, scallops, shrimp, squid, etc.)	○	○	○	○
Soy	○	○	○	○
Tree nuts (walnuts, cashews, etc.)	○	○	○	○
Wheat	○	○	○	○
Other (please describe in the next question)	○	○	○	○

What **other food(s)** has EVER caused any **allergic reactions** in your child?

What **medication(s)** has EVER caused any **allergic reactions** in your child?

What **other allergen(s)** has EVER caused any **allergic reactions** in your child?

[An allergen is a substance that causes an allergic reaction]

Questions about your baby's health Part 3

How many times has your child **experienced** the following **health conditions** and how many times has he/she been **diagnosed** with them by a health care provider in the past 6 months?

	# of times experienced	# of times diagnosed
Chickenpox/Varicella		
Constipation, chronic (stools that are hard, dry and painful to pass)		
Cradle cap		
Croup (inflammation of the voice box and windpipe)		
Diarrhea, chronic (stools that are more frequent or looser in consistency than your child's regular stools)		
Ear infection (count each episode, if both ears were infected in a single episode, count as 1)		
Fever of 103ºF or higher (count each episode, if fever lasted 3 days, count as 1)		
Fifth disease		
Hand-Foot-Mouth disease/Coxsackie virus		
Hay fever		
Head lice		
Heat rash		
Heat stroke		
Hives		
Impetigo		
Influenza/flu		
Measles		
Mumps		
Oral thrush		
Pinkeye		
Pinworms		
Pneumonia		
Ringworm		
Roseola		
RSV/Respiratory infection/bronchitis		
Rubella		

	# of times experienced	# of times diagnosed
Scabies		
Seizure		
Sinus infection/sinusitis		
Strep throat		
Thrush		
Tonsilitis		
Urinary tract infection		
Vomiting/gastroenteritis/norovirus/rotavirus		
Whooping cough		
Yeast diaper rash		

Has your child **experienced** any **other illness(es)** in the past 6 months that were not listed in previous questions?

*[**Exclude** common cold, runny nose, low-grade fever, and cough]*

○ Yes

○ No

○ Unsure

○ Refuse

How many **other illness(es)** has your child **experienced** in the past 6 months that were not listed in previous questions?

*[**Exclude** common cold, runny nose, low-grade fever, and cough]*

What **other illness(es)** has your child **experienced** in the past 6 months?

*[**Exclude** common cold, runny nose, low-grade fever, and cough]*

Other illness #1

What **other illness(es)** has your child **experienced** in the past 6 months?

*[**Exclude** common cold, runny nose, low-grade fever, and cough]*

Other illness #2

What **other illness(es)** has your child **experienced** in the past 6 months?

*[**Exclude** common cold, runny nose, low-grade fever, and cough]*

Other illness #3

What **other illness(es)** has your child **experienced** in the past 6 months?

*[**Exclude** common cold, runny nose, low-grade fever, and cough]*

Other illness #4

What **other illness(es)** has your child **experienced** in the past 6 months?

*[**Exclude** common cold, runny nose, low-grade fever, and cough]*

Other illness #5

What **other illness(es)** has your child **experienced** in the past 6 months?

*[**Exclude** common cold, runny nose, low-grade fever, and cough]*

Other illness #6

What **other illness(es)** has your child **experienced** in the past 6 months?

*[**Exclude** common cold, runny nose, low-grade fever, and cough]*

Other illness #7

What **other illness(es)** has your child **experienced** in the past 6 months?

*[**Exclude** common cold, runny nose, low-grade fever, and cough]*

Other illness #8

What **other illness(es)** has your child **experienced** in the past 6 months?

*[**Exclude** common cold, runny nose, low-grade fever, and cough]*

Other illness #9

What **other illness(es)** has your baby **experienced** in the past 6 months?

*[**Exclude** common cold, runny nose, low-grade fever, and cough]*

Other illness #10

Questions about sick doctor visits, hospitalizations, surgeries

Has your child had any **sick doctor visits** in the past 6 months?

[A sick-child doctor visit is an appointment the parent makes when the child is not feeling well]

- ○ Yes
- ○ No
- ○ Unsure
- ○ Refuse

How many **sick doctor visits** has your child had in the past 6 months?

What were the reasons for your child's **sick doctor visit(s)**?

Sick doctor visit #1

What were the reasons for your child's **sick doctor visit(s)**?

Sick doctor visit #2

What were the reasons for your child's **sick doctor visit(s)**?

Sick doctor visit #3

What were the reasons for your child's **sick doctor visit(s)**?

Sick doctor visit #4

What were the reasons for your child's **sick doctor visit(s)**?

Sick doctor visit #5

What were the reasons for your child's **sick doctor visit(s)**?

Sick doctor visit #6

What were the reasons for your child's **sick doctor visit(s)**?

Sick doctor visit #7

What were the reasons for your child's **sick doctor visit(s)**?

Sick doctor visit #8

What were the reasons for your child’s **sick doctor visit(s)**?

Sick doctor visit #9

What were the reasons for your child’s **sick doctor visit(s)**?

Sick doctor visit #10

What were the reasons for your child’s **sick doctor visit(s)**?

Sick doctor visit #11

What were the reasons for your child’s **sick doctor visit(s)**?

Sick doctor visit #12

Has your child had any **surgeries** in the past 6 months?

- ○ Yes
- ○ No
- ○ Unsure
- ○ Refuse

How many **surgeries** has your child had in the past 6 months?

What were the **surgery(s)** and the **reason(s)**?

	Surgery Name	Reason
Surgery #1		

What were the **surgery(s)** and the **reason(s)**?

	Surgery Name	Reason
Surgery #2		

What were the **surgery(s)** and the **reason(s)**?

	Surgery Name	Reason
Surgery #3		

What were the **surgery(s)** and the **reason(s)**?

	Surgery Name	Reason
Surgery #4		

What were the **surgery(s)** and the **reason(s)**?

	Surgery Name	Reason
Surgery #5		

What were the **surgery(s)** and the **reason(s)**?

	Surgery Name	Reason
Surgery #6		

What were the **surgery(s)** and the **reason(s)**?

	Surgery Name	Reason

	Surgery Name	Reason
Surgery #7		

What were the **surgery(s)** and the **reason(s)**?

	Surgery Name	Reason
Surgery #8		

What were the **surgery(s)** and the **reason(s)**?

	Surgery Name	Reason
Surgery #9		

What were the **surgery(s)** and the **reason(s)**?

	Surgery Name	Reason
Surgery #10		

What were the **surgery(s)** and the **reason(s)**?

	Surgery Name	Reason
Surgery #11		

What were the **surgery(s)** and the **reason(s)**?

	Surgery Name	Reason

	Surgery Name	Reason
Surgery #12		

Has your child been **hospitalized** in the past 6 months?

- ○ Yes
- ○ No
- ○ Unsure
- ○ Refuse

How many times has your child been **hospitalized** in the past 6 months?

What were the reasons for your child's **hospitalization(s)**?

Hospitalization #1

What were the reasons for your child's **hospitalization(s)**?

Hospitalization #2

What were the reasons for your child's **hospitalization(s)**?

Hospitalization #3

What were the reasons for your child's **hospitalization(s)**?

Hospitalization #4

What were the reasons for your child's **hospitalization(s)**?

Hospitalization #5

What were the reasons for your child's **hospitalization(s)**?

Hospitalization #6

What were the reasons for your child's **hospitalization(s)**?

Hospitalization #7

What were the reasons for your child's **hospitalization(s)**?

Hospitalization #8

What were the reasons for your child's **hospitalization(s)**?

Hospitalization #9

What were the reasons for your child's **hospitalization(s)**?

Hospitalization #10

What were the reasons for your child's **hospitalization(s)**?

Hospitalization #11

What were the reasons for your child's **hospitalization(s)**?

Hospitalization #12

Questions about antibiotics and probiotics

Has your child taken any **oral and/or IV antibiotics** at any time in the past 6 months?

[For this question, we are not asking about topical antibiotic ointments]

- Yes
- No
- Unsure
- Refuse

How many courses of **oral and/or IV antibiotics** has your child taken in the past 6 months?

Please list all of the **oral or IV antibiotics** your child has taken in the past 6 months, including the number of days your child took them and reasons he/she took antibiotics.

	Antibiotic Name<br><br>Brand Name	Was it Oral or IV?	Number of days antibiotic was taken	Reason for antibiotic<br><br>Reason
Antibiotic #1				

Please list all of the **oral or IV antibiotics** your child has taken in the past 6 months, including the number of days your child took them and reasons he/she took antibiotics.

	Antibiotic Name	Was it Oral or IV?	Number of days antibiotic was taken	Reason for antibiotic

	Brand Name			Reason
	Antibiotic Name<br><br>Brand Name	Was it Oral or IV?	Number of days antibiotic was taken	Reason for antibiotic<br><br>Reason
Antibiotic #2				

Please list all of the **oral or IV antibiotics** your child has taken in the past 6 months, including the number of days your child took them and reasons he/she took antibiotics.

	Antibiotic Name<br><br>Brand Name	Was it Oral or IV?	Number of days antibiotic was taken	Reason for antibiotic<br><br>Reason
Antibiotic #3				

Please list all of the **oral or IV antibiotics** your child has taken in the past 6 months, including the number of days your child took them and reasons he/she took antibiotics.

	Antibiotic Name<br><br>Brand Name	Was it Oral or IV?	Number of days antibiotic was taken	Reason for antibiotic<br><br>Reason
Antibiotic #4				

Please list all of the **oral or IV antibiotics** your child has taken in the past 6 months, including the number of days your child took them and reasons he/she took antibiotics.

	Antibiotic Name<br><br>Brand Name	Was it Oral or IV?	Number of days antibiotic was taken	Reason for antibiotic<br><br>Reason
Antibiotic #5				

Please list all of the **oral or IV antibiotics** your child has taken in the past 6 months, including the number of days your child took them and reasons he/she took antibiotics.

	Antibiotic Name<br><br>Brand Name	Was it Oral or IV?	Number of days antibiotic was taken	Reason for antibiotic<br><br>Reason
Antibiotic #6				

Please list all of the **oral or IV antibiotics** your child has taken in the past 6 months, including the number of days your child took them and reasons he/she took antibiotics.

	Antibiotic Name<br><br>Brand Name	Was it Oral or IV?	Number of days antibiotic was taken	Reason for antibiotic<br><br>Reason
Antibiotic #7				

Please list all of the **oral or IV antibiotics** your child has taken in the past 6 months, including the number of days your child took them and reasons he/she took antibiotics.

	Antibiotic Name<br><br>Brand Name	Was it Oral or IV?	Number of days antibiotic was taken	Reason for antibiotic<br><br>Reason
Antibiotic #8				

Please list all of the **oral or IV antibiotics** your child has taken in the past 6 months, including the number of days your child took them and reasons he/she took antibiotics.

	Antibiotic Name	Was it Oral or IV?	Number of days antibiotic was taken	Reason for antibiotic

	Brand Name<br>Antibiotic Name	Was it Oral or IV?	Number of days antibiotic was taken	Reason<br>Reason for antibiotic
	Brand Name			Reason
Antibiotic #9				

Please list all of the **oral or IV antibiotics** your child has taken in the past 6 months, including the number of days your child took them and reasons he/she took antibiotics.

	Antibiotic Name<br>Brand Name	Was it Oral or IV?	Number of days antibiotic was taken	Reason for antibiotic<br>Reason
Antibiotic #10				

Please list all of the **oral or IV antibiotics** your child has taken in the past 6 months, including the number of days your child took them and reasons he/she took antibiotics.

	Antibiotic Name<br>Brand Name	Was it Oral or IV?	Number of days antibiotic was taken	Reason for antibiotic<br>Reason
Antibiotic #11				

Please list all of the **oral or IV antibiotics** your child has taken in the past 6 months, including the number of days your child took them and reasons he/she took antibiotics.

	Antibiotic Name<br>Brand Name	Was it Oral or IV?	Number of days antibiotic was taken	Reason for antibiotic<br>Reason

	Antibiotic Name<br><br>Brand Name	Was it Oral or IV?	Number of days antibiotic was taken	Reason for antibiotic<br><br>Reason
Antibiotic #12				

<u>Excluding</u> yogurt, has your child taken any **probiotic supplement products** in the <u>past 6 months</u>?

[Probiotics are dietary supplements containing bacteria that are similar to beneficial bacteria found in the human gut]

- ○ Yes
- ○ No
- ○ Unsure
- ○ Refuse

Thinking about the **<u>past month</u>**, how many different **probiotic supplement products** has your child taken?

If your child took any **probiotics supplements** anytime in the **<u>past month</u>**, please list all of the brand and product names and how often your child took them.

[For this question, we are not asking about the dose or amount of probiotics your child took but the number of times your child took any amount. Please use your best guess]

[For example, Brand Name: Natren; Product Name: Life Start]

	Probiotic Brand & Product		# of times taken in the past month
	Brand name	Product name	
Probiotic #1			

If your child took any **probiotics supplements** anytime in the **past month**, please list all of the brand and product names and how often your child took them.

[For this question, we are not asking about the dose or amount of probiotics your child took but the number of times your child took any amount. Please use your best guess]

[For example, Brand Name: Natren; Product Name: Life Start]

	Probiotic Brand & Product		# of times taken in the past month
	Brand name	Product name	
Probiotic #2			

If your child took any **probiotics supplements** anytime in the **past month**, please list all of the brand and product names and how often your child took them.

[For this question, we are not asking about the dose or amount of probiotics your child took but the number of times your child took any amount. Please use your best guess]

[For example, Brand Name: Natren; Product Name: Life Start]

	Probiotic Brand & Product		# of times taken in the past month
	Brand name	Product name	
Probiotic #3			

If your child took any **probiotics supplements** anytime in the **past month**, please list all of the brand and product names and how often your child took them.

[For this question, we are not asking about the dose or amount of probiotics your child took but the number of times your child took any amount. Please use your best guess]

[For example, Brand Name: Natren; Product Name: Life Start]

	Probiotic Brand & Product		# of times taken in the past month
	Brand name	Product name	
Probiotic #4			

Questions about the feeding patterns of your baby enrolled in this study

Questions about the feeding patterns of your child enrolled in this study

Has your child consumed any amount of your **breast milk** (either at the breast or by bottle) in the past month?

- ○ Yes
- ○ No
- ○ Unsure
- ○ Refuse

How did your child mostly consume **your breast milk** in the **past 7 days**?

[This questions is not asking about donated breast milk]

- ○ Mostly at the breast
- ○ Mostly with a bottle
- ○ About half at the breast and half with a bottle
- ○ My child did not consume my breast milk in the past 7 days
- ○ Unsure
- ○ Refuse

Thinking about the **past 7 days**, about how many **bottles of your breast milk** has your child consumed **per day**?

[For this question, we are not asking about the amount of your breast milk in each bottle]

About how many **ounces of your breast milk** did your child drink during a **typical bottle feeding** in the **past 7 days**?

[For this question, we are asking about how much breast milk your child actually drank NOT how much breast milk was prepared in the bottle]

Thinking about the **past 7 days**, about how many times have you **breastfed your** child at the breast **per day**?

Has your child consumed any amount of **donor breast milk** in the **past 7 days**?

[Donor breast milk is breast milk from anyone other than you, fed by a bottle]

- ○ Yes
- ○ No
- ○ Unsure
- ○ Refuse

Thinking about the **past 7 days**, about how many **bottles of donor breast milk** did your child drink **per day**?

[For this question, we are not asking about the amount of donor breast milk in each bottle]

About how many **ounces of donor breast milk** did your child drink during a **typical bottle feeding** in the **past 7 days**?

[For this question, we are asking about how much donor breast milk your child actually drank not how much was in the bottle]

Questions about your baby’s Environment

Questions about your child's environment

Has your child attended any formal part-time or full-time **daycare programs** in the past month?

[Include institutional and at-home daycares in which other babies are enrolled. Do not include Mommy-and-Me classes]

- ○ Yes
- ○ No
- ○ Unsure
- ○ Refuse

Approximately how old was your child when he/she was first enrolled into **daycare**? (age of child in **months**)

[▾]

How many **days per week** did your child spend in any **daycare** in the past month?

- ○ 1-2 days/week
- ○ 3-4 days/week
- ○ 5 days/week
- ○ Unsure
- ○ Refuse

How many **hours per day** did your child spend in any **daycare** in the past month?

- ○ 1-2 hours/day
- ○ 2-4 hours/day
- ○ 4-6 hours/day
- ○ More than 6 hours/day
- ○ Unsure
- ○ Refuse

How many children, **excluding** your child enrolled in this study, currently live with you?

What are the **ages** of all of the children that currently live with you, **excluding** your child enrolled in this study?

	Years	Months
Child #1		

What are the **ages** of all of the children that currently live with you, **excluding** your child enrolled in this study?

	Years	Months
Child #2		

What are the **ages** of all of the children that currently live with you, **excluding** your child enrolled in this study?

	Years	Months
Child #3		

What are the **ages** of all of the children that currently live with you, **excluding** your child enrolled in this study?

	Years	Months
Child #4		

What are the **ages** of all of the children that currently live with you, **excluding** your child enrolled in this study?

	Years	Months
Child #5		

What are the **ages** of all of the children that currently live with you, **excluding** your child enrolled in this study?

	Years	Months
Child #6		

What are the **ages** of all of the children that currently live with you, **excluding** your child enrolled in this study?

	Years	Months
Child #7		

What are the **ages** of all of the children that currently live with you, **excluding** your child enrolled in this study?

	Years	Months
Child #8		

What are the **ages** of all of the children that currently live with you, **excluding** your child enrolled in this study?

	Years	Months
Child #9		

What are the **ages** of all of the children that currently live with you, **excluding** your child enrolled in this study?

	Years	Months
Child #10		

Are you currently **pregnant**?

- ○ Yes
- ○ No
- ○ Unsure
- ○ Refuse

What is your **due date**?

Month

Day

Year

Open ended comment

If there is anything else you would like to share regarding your baby's well-being please add your comments below. Otherwise, write "none".

End of Questionnaire

End of Questionnaire

Please make sure your answers are complete and review them if necessary.

Click the **next button** once you have completed the survey. Once you click the next button, you will not be able to go back to see your answers.

Survey Powered By Qualtrics
