# Supplementary figures and images for: Early probiotic supplementation with B. infantis in breastfed infants leads to persistent colonization at 1 year
Source: Pediatr Res. 2021 Mar 24;91(3):627–36. doi: 10.1038/s41390-020-01350-0 (PMC8460680; doi:10.1038/s41390-020-01350-0)

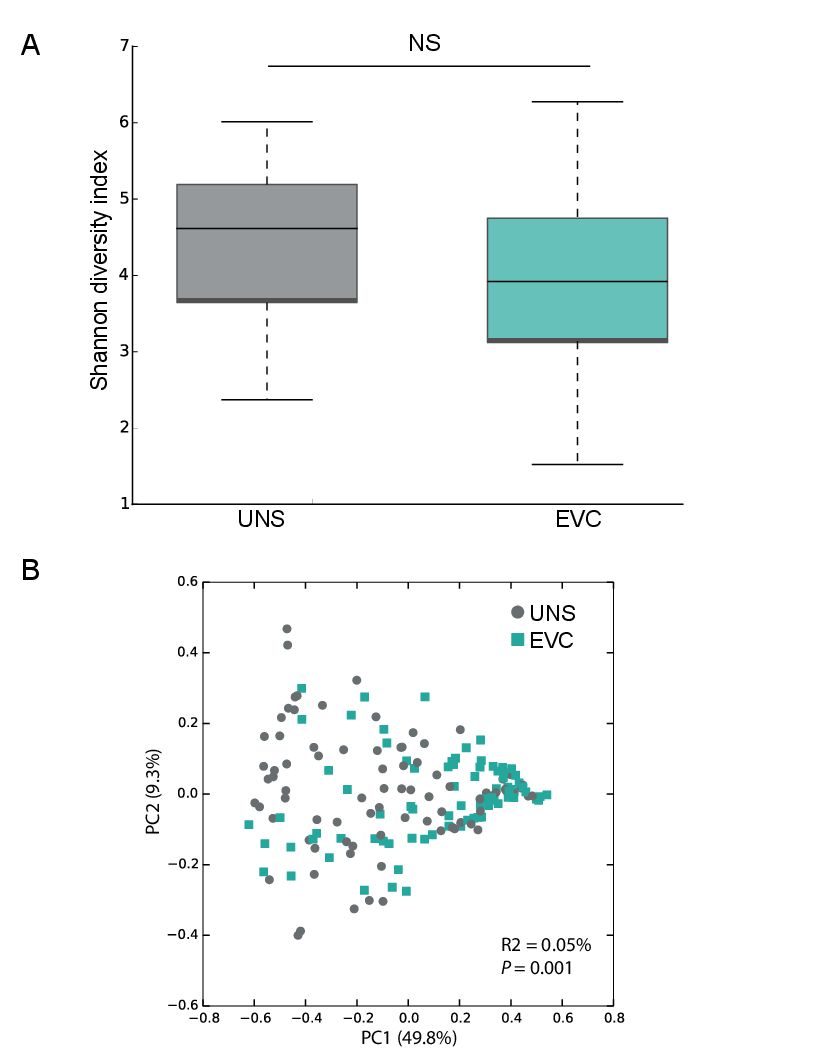

Supplement: Supplementary file 3 — Supplementary Figure 3 [file 41390_2020_1350_MOESM3_ESM.jpg]
